# Recent advances of IDH1 mutant inhibitor in cancer therapy

**DOI:** 10.3389/fphar.2022.982424

**Published:** 2022-08-24

**Authors:** Wangqi Tian, Weitong Zhang, Yifan Wang, Ruyi Jin, Yuwei Wang, Hui Guo, Yuping Tang, Xiaojun Yao

**Affiliations:** ^1^ College of Pharmacy, Shaanxi University of Chinese Medicine, Xianyang, Shaanxi, China; ^2^ State Key Laboratory of Quality Research in Chinese Medicine, Macau University of Science and Technology, Taipa, China

**Keywords:** mIDH1 inhibitors, isocitrate dehydrogenase mutation, hypermethylation, 2-HG, natural product

## Abstract

Isocitrate dehydrogenase (IDH) is the key metabolic enzyme that catalyzes the conversion of isocitrate to α-ketoglutarate (α-KG). Two main types of IDH1 and IDH2 are present in humans. In recent years, mutations in IDH have been observed in several tumors, including glioma, acute myeloid leukemia, and chondrosarcoma. Among them, the frequency of IDH1 mutations is higher than IDH2. IDH1 mutations have been shown to increase the conversion of α-KG to 2-hydroxyglutarate (2-HG). IDH1 mutation-mediated accumulation of 2-HG leads to epigenetic dysregulation, altering gene expression, and impairing cell differentiation. A rapidly emerging therapeutic approach is through the development of small molecule inhibitors targeting mutant IDH1 (mIDH1), as evidenced by the recently approved of the first selective IDH1 mutant inhibitor AG-120 (ivosidenib) for the treatment of IDH1-mutated AML. This review will focus on mIDH1 as a therapeutic target and provide an update on IDH1 mutant inhibitors in development and clinical trials.

## Introduction

The tricarboxylic acid cycle exists in the metabolic process of most organisms, which is a series of enzymatic reactions that help organisms oxidize sugar or other substances to obtain energy (Scagliola et al., 2020). As a rate-limiting enzyme involved in the tricarboxylic acid cycle, the isocitrate dehydrogenase (IDH) family plays an important role in catalyzing the conversion of ketoglutarate to hydroxyglutarate. The IDH family includes three isozymes, IDH1, IDH2, and IDH3. IDH1 is present in the cytoplasm, while IDH2 and IDH3 are present in the mitochondrion (Yang et al., 2010). IDH1 and IDH2 are the isoforms implicated in tumourigenesis. The maximum mutation’s frequency of IDH1 is more than 90% in gliomas, while the mutation’s frequency of IDH2 is less than 5%. Thus, IDH1 has attracted a lot of attention as a hot target for research. A large number of research teams are committed to seeking more efficient inhibitors to achieve targeted therapy for diseases caused by IDH1 mutations. This article describes the basic structure of IDH1, the possible diseases caused by IDH1 mutations, and summarizes the effective inhibitors discovered so far. This provides a reference for the further development of IDH1 mutant inhibitors.

**FIGURE 1:**
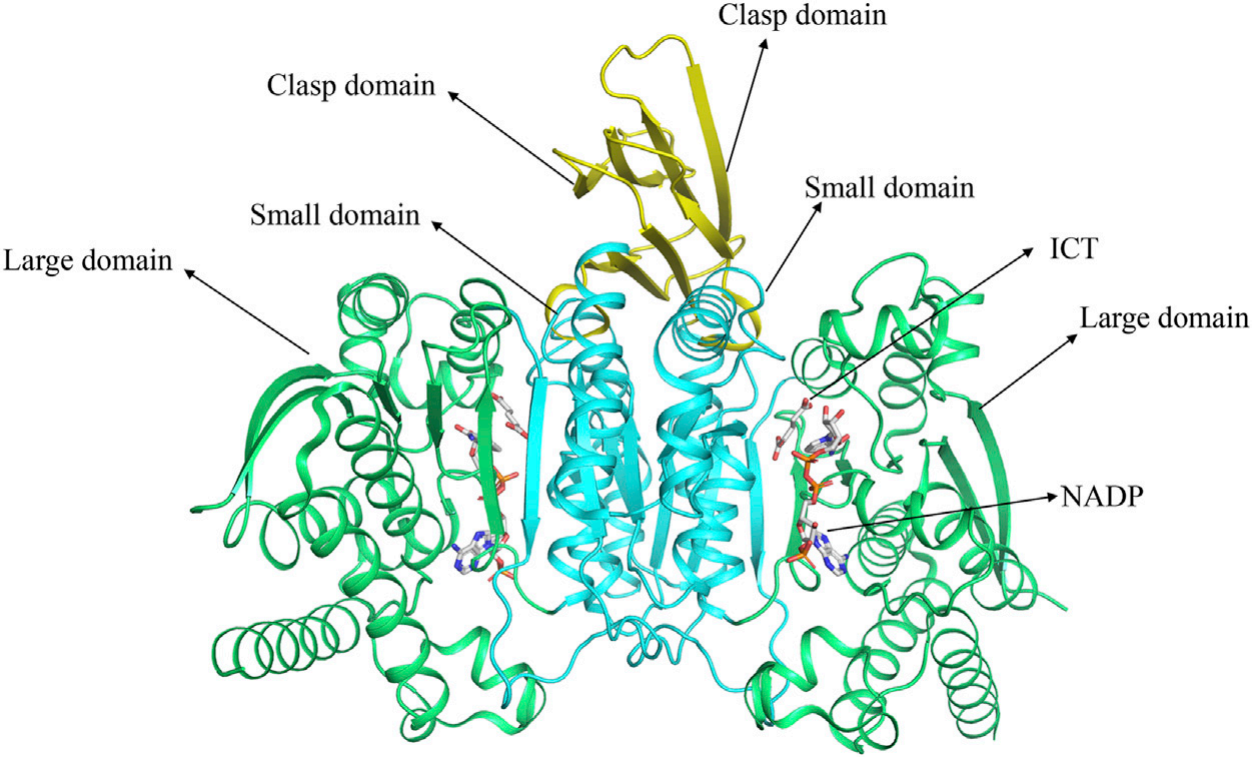
The structure of IDH1 and the distribution of various domains.

The gene encoding human IDH1 is located on chromosome 2q33.3 and has a full length of 18,854 nucleotides that span 18.9 kb. Its mRNA is 2339 bp in length and has 10 exons. It encodes 414 amino acids in the IDH1 enzyme (Zhu et al., 2014). Human IDH1 is considered to be NADP-IDHs (Chen and Gadal, 1990). NADP-IDH1 exists in two structures in organisms: monomer and homodimer (Imabayashi et al., 2006). Human IDH1 belongs to homodimer IDH. IDH1 is consist of three domains: A large domain (Domain A), a small domain (Domain C), and a clasp domain (Domain B) (Figure 1) (Hurley et al., 1989; Yasutake et al., 2002). Domain A consists of five β-strands (βA-βE) and eight α-helices (αA-αD and αJ-αM). Domain C consists of seven β-strands (βF-βL) and four α-helices (αF-αI). Domain B has the simplest structure, including two β-strands (βM-βN) and one α-helix (αE). Domain A and Domain C are connected by 12 parallel β-sheets in the middle, which have own hydrophobic core, which makes them separated by two pockets. The larger one is a hydrophilic “active pocket” and the smaller is a hydrophobic “back pocket”. The “active pocket” is the IDH catalytic site, which contains two binding sites: one is the substrate-binding site and the other is the cofactor-binding site.

## Isocitrate dehydrogenase1 mutation and 2-hydroxyglutarate involvement in epigenetic regulation

### Molecular mechanism of isocitrate dehydrogenase1 mutation

**FIGURE 2:**
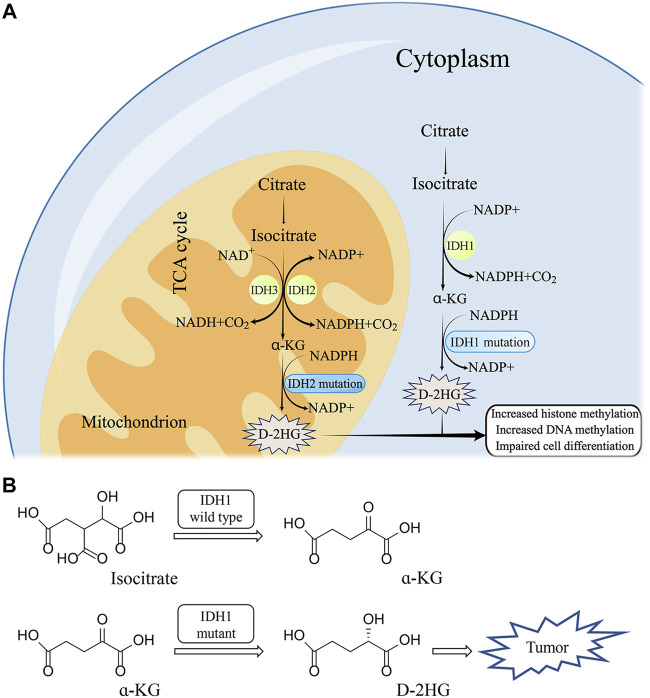
The molecular mechanism of IDH1 mutation **(A)** and corresponding structure of substrate **(B)**.

Under the action of Mg^2+^, IDH1 can oxidatively decarboxylate isocitrate to form α-KG (Liu et al., 2021). Generally, Human cells produce low concentrations of D-2HG and L-2HG once catalyzed by normal enzymes (Losman and Kaelin, 2013). D-2HG and L-2HG can be converted into α-KG in time by 2-hydroxyglutarate dehydrogenase (L-2HGDH and D-2HGDH) so as not to accumulate (Ye et al., 2018) (Figure 2A). However, when IDH1 is mutated, it can promote the conversion of α-KG to D-2HG, resulting in excessive accumulation of D-2HG in mutated tumor cells (Han, et al., 2020) (Figure 2B). The IDH1 mutation is the substitution of arginine 132 by other amino acids, including R132 H/C/L/S (Golub, et al., 2019). Particularly, R132H and R132C are the most common mutation types, with mutation’s frequency ranging from 4.3% to 33% (Kim, et al., 2020).

### 2-hydroxyglutarate is involved in epigenetic regulation

The massive accumulation of 2-HG in the body after IDH1 mutation will be involved in epigenetic regulation. The structural similarity leads to 2-HG acting as an antagonist of α-KG, thereby inhibiting the activity of various dioxygenases. Chowdhury et al. (2011) found that histone demethylases KDM4A, KDM4C, and KDM2A are all sensitive to 2-HG. 2-HG can inhibit TET protein activity of DNA demethylation enzyme. Yang et al. (2017) found that 2-HG promotes DNA methyltransferase 1 (DNMT1) binding to specific DNA regions, causing oncogene promoter hypermethylation and downregulation of expression, thereby promoting tumor development. 2-HG also inhibits the activity of prolyl hydroxylase domain-containing proteins (PHDs) and promotes the proliferation of human cancer cells (Koivunen et al., 2012). 2-HG can impair collagen maturation by inhibiting PLOD1-3 and C-P4H1-3, resulting in the abnormal basement membrane and promoting glioma cell invasion and metastasis (Su et al., 2018). 2-HG affects apoptosis by inhibiting the activity of mitochondrial complex IV. 2-HG also can affect intracellular calcium homeostasis and promote the generation of reactive oxygen species (ROS). 2-HG can inhibit the activity of ATP synthase in mitochondria and key enzymes SDH and FH in the tricarboxylic acid cycle, resulting in disorder of cellular metabolism. 2-HG has the same inhibitory effect on AlkB family proteins (Wang et al., 2015), which are involved in the repair of DNA damage caused by alkylating agents. It has been shown that 2-HG can significantly reduce the rate of DNA repair in mutant IDH cells, resulting in increased DNA damage. 2-HG also prevents cell differentiation and reduces the expression of the tumor suppressor gene p53 through epigenetic regulation (Dang and Su, 2017; Jiang et al., 2018; Madala et al., 2018). Some researchers have attempted to exert anti-tumor effects through modulating epigenetic factors, similar to the development of PRMT5 inhibitors (Chen et al., 2021). IDH1 mutation-mediated diseases may also be treated with this approach.

## Related diseases induced by mutant isocitrate dehydrogenase

IDH1 mutations were first identified by exome sequencing of colon tumors and glioblastoma multiforme (Sjöblom et al., 2006; Parsons et al., 2008). It was found that IDH mutations mainly occurred at different arginine residues in IDH1 R132. A large number of studies have found that IDH1 mutations are associated with the occurrence and development of various cancers.

### Acute myeloid leukemia

AML is a malignant clonal disease derived from hematopoietic stem cells (Lu et al., 2019). Patients usually suffer from anemia, bleeding, and infections due to a decrease in the number of white blood cells. At present, researchers frequently detect epigenetic gene mutations in the onset of AML. In AML, the mutant frequency of IDH1/2 is approximately 20% (Duchmann et al., 2021; McMurry et al., 2021). It is generally believed that the pathogenesis of IDH-induced AML may be related to the hypermethylation of the entire genome after IDH mutation (Jingtao et al., 2021). Clinical treatment of AML is usually based on chemotherapy, targeted therapy, and hematopoietic stem cell transplantation (Döhner et al., 2021). In the past 2 years, the FDA approved eight new drugs for the treatment of AML, including Midostaurin, Gilteritinib, CPX-351, Gemtuzumab Ozogamicin, anti-CD33 monoclonal antibody, Ivosidenib, enasidenib, Venetoclax (Fiorentini et al., 2020). Among them, Ivosidenib is the only marketed mIDH1 inhibitor.

### Chondrosarcoma

Chondrosarcoma occurs mostly in the long bones of the extremities or the pelvis and is one of the most common malignant bone tumors. The tumor grows slowly, and the rate of recurrence and metastasis is low. Therefore, surgical resection is often the main clinical practice. A large number of studies have found that about 38%–86% of chondrosarcoma cases have IDH mutations (Azzi et al., 2014). Nissree *et al.* found that the level of 2-HG in chondrosarcoma caused by IDH1 mutations was significantly increased (Mohammad et al., 2020).

### Glioblastoma

According to the molecular mechanism, glioblastoma can be divided into primary and secondary. Because of its special disease site and space-occupying effect, patients will have language barriers, movement disorders, or comprehension disorders (Weller et al., 2015). The high degree of invasiveness and high fatality rate makes the clinical treatment effect of the disease unsatisfactory. Clinically, the effect of targeted drugs in the treatment of glioma is not significant (Chen et al., 2017). Therefore, the clinical treatment of glioma mainly relies on traditional methods, such as surgery, chemotherapy, and radiotherapy (Xu et al., 2020). Studies have found that the mutation’s frequency of IDH1 genes in oligodendroglioma, astroglioma, and secondary glioblastoma is as high as 60%–80% (Han et al., 2020), but in primary glioblastoma almost absent in the tumor.

## Isocitrate dehydrogenase mutant inhibitors

To date, more than two dozen small-molecule compounds have been reported as mIDH1 inhibitors. We have summarized the currently published mIDH1 inhibitors and briefly described their mechanism of action based on molecular docking (see Table 1).

**TABLE 1:** The reported mIDH1 inhibitors and key residues.

mIDH1 inhibitors	Target protein	Key residues	References
Indane analogues	IDH1	ILE128, LEU120, ALA111, TRP124	Zheng et al. (2017)
IDH305	IDH1	ILE128, LEU120, SER278, VAL121	Cho et al. (2017)
AG-881	IDH1/2	GLN277	Konteatis et al. (2020)
BAY1436032	IDH1	SER280, HIS232	Gokul and Rajanikant, (2017)
FT-2102	IDH1	ILE128, TRP267, ARG109, ASP279	Lin et al. (2019)
VVS	IDH1	ASP279	Deng et al. (2015)
GSK-321	IDH1	Ile128, Pro127, Trp124, Arg119, Leu120	Okoye-Okafor et al. (2015)
DS-1001b	IDH1	Asp 275, Asp 279, Asp 252	Machida et al. (2020)
3-aryl-4-indolyl-maleimides	IDH1	Ile128, Ala111, Arg119, Trp124, Val255, Met259, Leu120	Hu et al. (2018); Liu et al. (2019)
SYC-435	IDH1	Arg100, Ser94, Thr77, Asn96, Arg109	Liu et al. (2014); Zheng et al. (2013)
Compound 13	IDH1	Thr77, Ser94, Asn96, Gly97, Arg100, Asn101, Arg109	Wu et al. (2015)
HMS-101	IDH1	Arg314, Asn328, Leu288, Gly310, Val312	Chaturvedi et al. (2020)
DC_H31	IDH1	Gln277, Ser280, Trp124, Tyr285, Trp267	Duan et al. (2019)
WM-17	IDH1	TRP124, ILE128, ALA258, TRP267	Zhang et al. (2021)
CRUK-MI	IDH1	Arg119, Tyr285, Val255, Met 259	Jones, et al. (2016)
ZX-06	IDH1	LEU120, ILE128, VAL281, ASP279	Zou, et al. (2018)
L806-0255	IDH1	SER287, ILE228, MET259	Wang et al., 2020)
V015-1671	IDH1	SER-278, ALA111, ILE128	Wang et al. (2020)
AQ-714/41674992	IDH1	ILE-128, ALA111	Wang et al. (2020)

### Phenyl-glycine compounds

**FIGURE 3:**
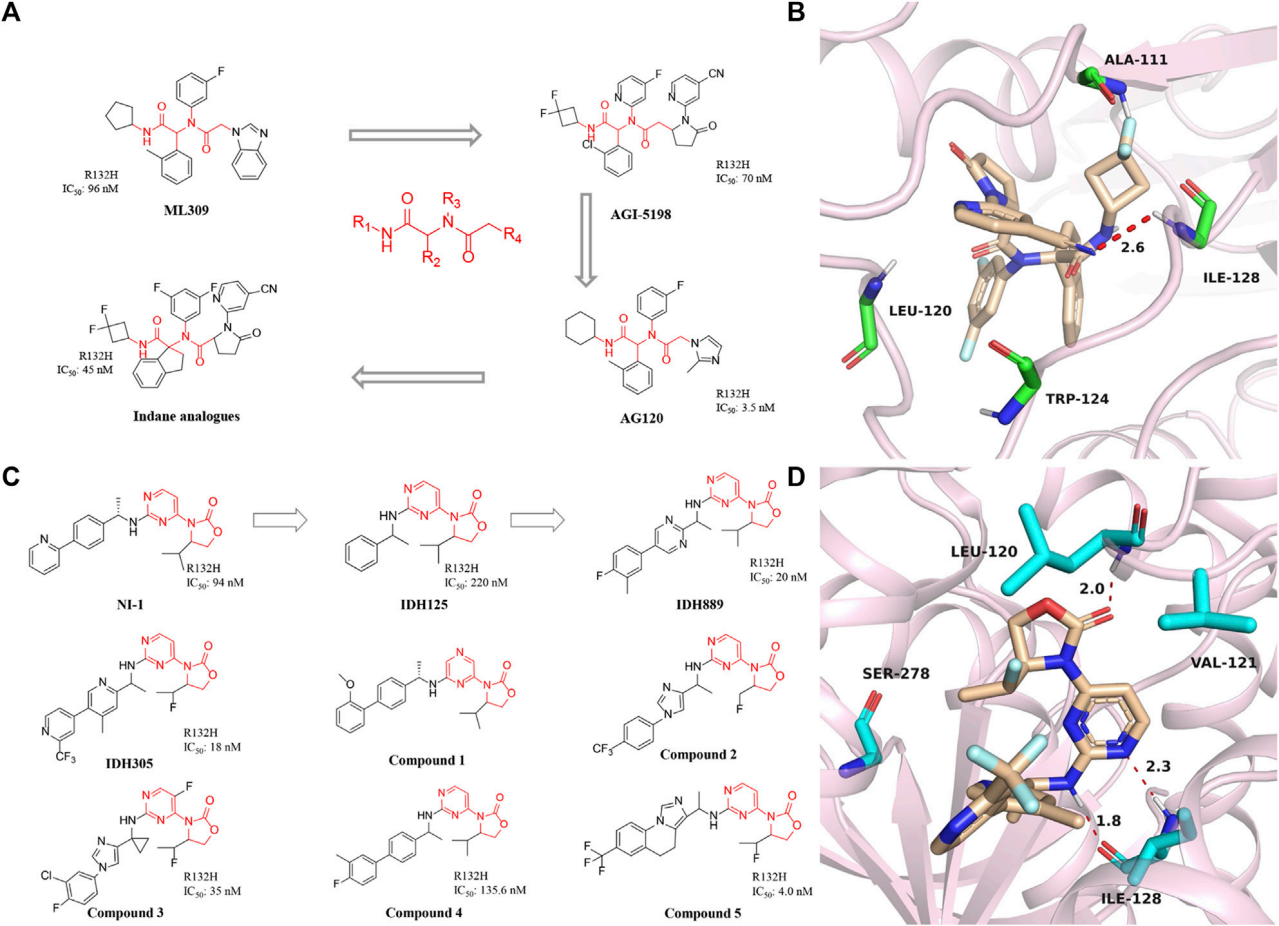
**(A)** The structure of phenyl-glycine compounds. **(B)** The binding mode of Indane analogs with mIDH1 R132H. **(C)** The structure of 3-pyrimidin-4-yl-oxazolidin-2-ones. **(D)** The binding mode of IDH305 with mIDH1 R132H.

Mindy et al. first reported the inhibitor targeting mIDH1 in 2012, ML309, which has a phenyl-glycine backbone (Figure 3A) (Davis et al., 2010). ML309 exhibits good selectivity between wild-type and R132H mutant IDH1, which can effectively reduce the production of cell-based 2-HG in the U87MG cell line (Gerecke et al, 2020). In addition, it was found that ML309 has better ADME properties *in vitro* and less toxicity with IC_50_ of 96 nM (Davis et al., 2014). There is currently no crystal structure indicating the specific binding mode of ML309 to mIDH1 R132H. But compared with WT IDH1, ML309 binds more easily to mIDH1 R132H, which may be related to the more open conformation of mIDH1 R132H (Merk et al., 2016). ML309 also has good water solubility and high stability in human plasma. *In vivo* PK experiments, ML309 has a relatively ideal C_max_ and a reasonable half-life time. Unfortunately, brain exposure to the substance has not been observed. Therefore, ML309 may not be the ideal option for the treatment of glioma.

Dan *et al.* obtained the compound AGI-5198 though optimizing the structure of ML309. AGI-5198 is a potent and selective mIDH1 inhibitor developed by Agios. The IC_50_ of AGI-5198 for IDH1 R132H/R132C mutation is 70 nM and 160 nM, respectively (Rohle et al., 2013). AGI-5198 exhibits anti-tumor effect on the TS603 glioma cell line and inhibits the production of 2-HG in a dose-dependent manner (Dowdy et al., 2020). AGI-5198 induces histone H3K9me3 demethylation while completely inhibiting the production of 2-HG. It shows a strong inhibitory effect in the xenograft model. The magnitude and duration of the inhibition of 2-HG in tumor cell correlate with free drug plasma concentration. Importantly, AGI-5198 inhibits the growth of mIDH1 glioma but has no significant inhibitory effect on IDH1 wild-type gliomas. AGI-5198 also has many disadvantages, such as being easily metabolized in the body and cleared out of the body, and poor druggability, thus limiting its further clinical application.

Based on AGI-5198, Agios designed the AG-120 and called it ivosidenib, which is the only oral small molecule targeting mIDH1 approved by the FDA for the treatment of AML. AG-120 is highly selective for IDH1 mutants but has no significant effect on IDH2 mutants (Popovici-Muller et al., 2012). *In vitro* tests, AG-120 exhibits excellent enzymatic activity, cellular activity, and stability of liver microsomes. PK studies demonstrated that AG-120 is rapidly absorbed after oral administration, with low systemic plasma clearance and a long half-life. An early clinical trial [NCT02074839] showed an overall response rate of 42% and a complete response rate of 22% in patients with mIDH1 AML treated with ivosidenib. In phase III clinical trials, the recommended dose of ivosidenib is 500 mg/d (Fan et al., 2020a; Stein et al., 2021). Based on the current clinical trial results, ivosidenib has become the first-line drug for the treatment of AML, providing AML patients with better treatment options and chances of survival (Norsworthy et al., 2019). In addition, the brain penetration rate of AG-120 in rats with intact blood-brain barrier was 4.1% (Popovici-Muller et al., 2018). Therefore, the researchers speculated that the brain penetration rate of ivosidenib may be higher in patients with a compromised blood-brain barrier, making it easier to reach therapeutic concentrations in patients. In this regard, a clinical study of this drug for the treatment of glioma has been carried out [NCT03343197]. In addition, a phase III clinical trial was used to evaluate the efficacy and safety in patients with intrahepatic cholangiocarcinoma [NCT02989857] (Zhu et al., 2021). The results showed that ivosidenib have significantly improved progression-free survival in patients compared with placebo, and the drug was well tolerated (Abou-Alfa et al., 2020). The treatment of chondrosarcoma with ivosidenib is currently in the phase I clinical stage. The patients received oral treatment for 28 consecutive days (100 mg twice daily to 1,200 mg once daily), and the plasma 2-HG levels of all patients decreased significantly to drop to the level range of healthy individuals. For patients with advanced mIDH1 chondrosarcoma, ivosidenib shows low toxicity and relatively ideal treatment results, which is expected to be further promoted in clinical (Fan et al., 2020b; Tabata et al., 2020).

In 2017, Shanghai Haihe Pharmaceutical modified the structure of the AG-120 molecule and named it indane analogs (Zheng et al., 2017). The newly engineered compound has IC_50_ of 45 nM for IDH1 R132H. But it is worth noting that the IC_50_ < 5 nM of this compound in the HT1080 cell line is lower than the 7.5 nM of AG-120. Besides, the indane analogs have more perfect PK properties. Molecular docking shows that the carbonyl group at the end of the indane analogs is immobilized in the active pocket through one critical hydrogen bond with Ala111 residue (Figure 3B) (PDB ID: 5TQH). In addition, the main chain NH of cyano pyridine combines with the two residues leu120 and Ile128 to form key hydrogen bonds. The indane moiety also forms π-π stacking with the indole ring of Trp124. These results demonstrate that existing potent inhibitors can be engineered to produce compounds that are less toxic or better metabolized.

### 3-Pyrimidin-4-yl-oxazolidin-2-ones

A reported patent numbered WO2013046136 A1 in 2014 (Figure 3C) (Caferro et al., 2015) describes a compound named NI-1, which indicates that NI-1 may be used to treat diseases with mutations in the IDH protein. Subsequently, IDH125 and IDH889 were reported successively (Levell et al., 2017). The researchers first obtained IDH125 through high-throughput screening. IDH125 has good selectivity and cellular activity, but the inhibition rate for mutant IDH1 is not strong enough. After that, IDH889 was obtained by modifying the structure of IDH125. IDH889 demonstrates brain penetration exposure and inhibitory activity of 2-HG in a mIDH1 xenograft mouse model.

In the same year, Novartis reported the compound IDH305, which is an oral inhibitor in Phase I clinical trials (Cho et al., 2017). The drug has IC_50_ of 18 nM and is nearly 200-fold more selective for mIDH1 R132H than wild-type. The X-ray structure of IDH305 bound to the homodimer mIDH1 R132H shows in Figure 3D (PDB ID: 6B0Z). The aminopyrimidine moiety forms a pair of hydrogen bonds with the backbone atoms of Ile128, while the carbonyl of the oxazolidinone forms a hydrogen bond with the backbone amide of Leu120. IDH305 demonstrates a hydrophobic collapse conformation in which the fluoroethyl is in van der Waals contact with the pyridine of the amine side chain. The bipyridine moiety extends out toward the dimer interface. The side chain of Ser278 is positioned in the vicinity of the ortho nitrogen of the pyridine ring, potentially forming a hydrogen bond. Preclinical tests have shown that IDH305 can significantly reduce the level of 2-HG in tumors. Based on the compound’s good activity and PK properties, researchers conducted the first clinical trial in 2016 [NCT02381886]. Unfortunately, there has been no new clinical research data in recent years. Besides, Tong et al. (2021) used the QSAR model to study 3-pyrimidin-4-yl-oxazolidin-2-one derivatives.

IDH305 is a class of selective mIDH1 inhibitors, but the clearance effect of this substance *in vivo* is not satisfactory (Zhao et al., 2018). The optimization work is carried out by researchers at Novartis Institutes for BioMedical Research. Based on not compromising other properties of the compound, it focuses on improving the clearance rate and the metabolic stability of the drug. Compound **1** was screened out, which has demonstrated brain penetration and excellent oral bioavailability in rodents. In a preclinical patient-derived IDH1 mutant xenograft tumor model study, compound **1** efficiently inhibited the production of the biomarker 2-HG.

In 2017, China Pharmaceutical University reported compound **2**, which shares the same backbone structure as IDH889 (Ma et al., 2017). But after modification, compound **2** can penetrate the blood-brain barrier, which is very important for the development of drugs for the central nervous system.

In 2018, a series of imidazole cyclopropyl anime analogs were reported by the University of Chinese Academy of Sciences (Zheng Q et al., 2018). The best of these compounds **3** shows potent inhibition of mIDH1 R132H enzymatic activity and the production of 2-HG, modest liver microsome stability, and PK properties in the mIDH1 HT1080 cell line. In 2019, compound **4** was synthesized and demonstrated to specifically inhibit IDH1 mutation-mediated reduction of α-KG to 2-HG (Jia et al., 2019). Compound 4 can reduce histone methylation levels, and induce hepatocyte differentiation and other characteristics. Besides, compound **5** with a similar backbone was designed and synthesized in 2019 (Cao et al., 2019). In addition to good inhibitory activity in terms of enzymatic activity, compound **5** can also break through the blood-brain barrier to reach the auction site.

### Triazine compounds

**FIGURE 4:**
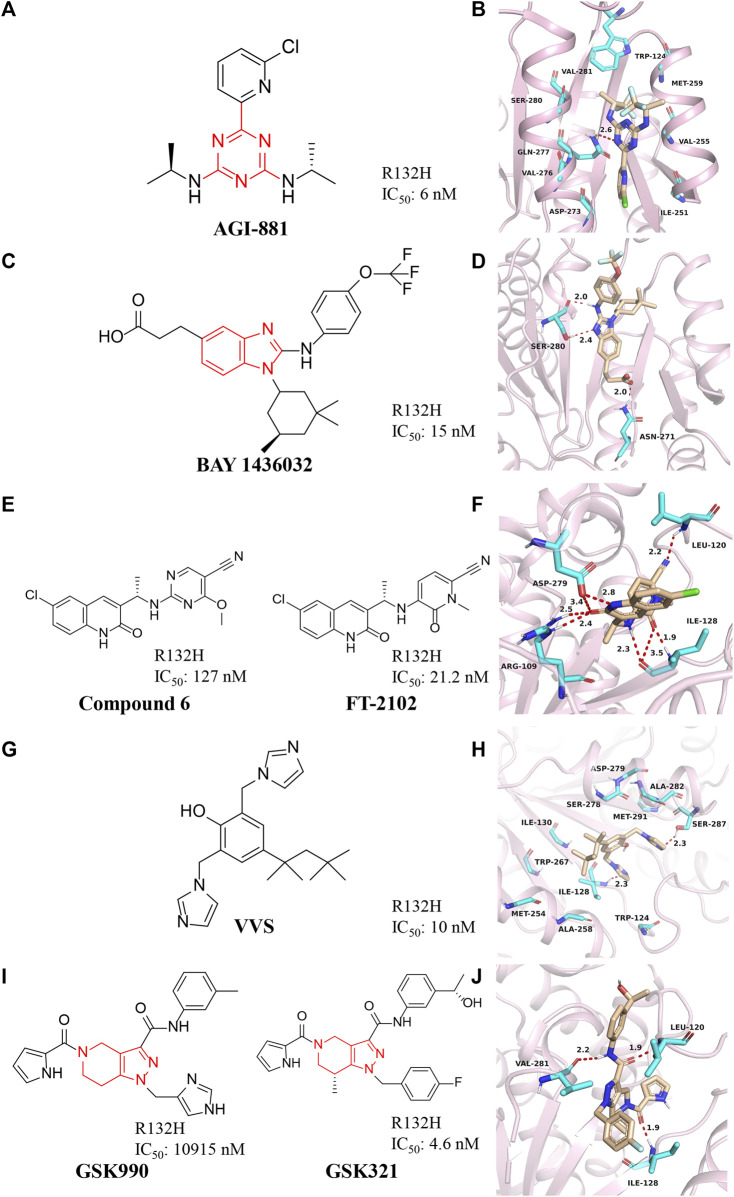
**(A,B)** The structure of AG-881 and binding mode with mIDH1. **(C,D)** The structure of BAY1436032 and binding mode with mIDH1. **(E,F)** The structure of compound 6 and binding mode with mIDH1. **(G,H)** The structure of VVS and binding mode with mIDH1. **(I,J)** The structure of GSK321 and binding mode with mIDH1.

AG-881 is a dual inhibitor developed by Agios against IDH1 and IDH2 mutations (Figure 4A) (Konteatis et al., 2020). The drug is effective against IDH1 R132C, IDH1 R132L, IDH1 R132H, and IDH1 R132S mutations (IC_50_ = 0.04–22 nM). AG-881 forms multiple hydrophilic and hydrophobic interactions with amino acids within the binding pocket, and most of these residues are conserved in mIDH1 (Figure 4B) (PDB ID: 6ADG) (Ma and Yun, 2018). AG-881 is in Phase I clinical trials (Radoul et al., 2021). Because of its high brain penetration, it is promising for the treatment of gliomas in the future (Konteatis et al., 2020). Currently, a Phase 1 trial of AG-881 in solid tumors including gliomas is underway [NCT02481154], and 93 patients are doing well (Mellinghoff et al., 2021). In addition, clinical trials for advanced hematological malignancies are ongoing [NCT02492737].

### Benzimidazole scaffold

BAY1436032 is a highly selective, potent, and highly bioavailable mIDH1 inhibitor developed by Bayer (Figure 4C) (Gokul and Rajanikant, 2017). The crystal structure shows that BAY1436032 does not bind to the active site but to an allosteric pocket located between the homodimeric mIDH1 R132H complex (Figure 4D) (PDB ID: 5LGE). The benzimidazole scaffold and the aniline nitrogen form two hydrogen bonds with Ser280, while the carboxyl group interacts *via* a salt bridge with His132 of mIDH1 R132H. The isopropyl phenyl and cyclohexyl groups insert into hydrophobic subpocket within the binding cavity, which is almost completely buried and shielded from the solvent. BAY1436032 potently inhibits the release of 2-HG in cells with IC_50_ of 15 nM against mutant IDH1 R132H protein. BAY1436032 can significantly prolong the survival time in mice transplanted with IDH1-mutated tumors (Wenger, et al., 2020). It is in the clinical phase I of the research stage (Chaturvedi et al., 2017). BAY1436032 is used for the treatment of AML [NCT03127735] and advanced solid tumors [NCT024746081], but there are no relevant clinical reports yet (Heuser et al., 2020; Wick et al., 2021).

### 1H-quinolin-2-one

Compound **6** with a quinolinone skeleton was found to have inhibitory effects as well in 2019 (Figure 4E), which exhibits good overall selectivity and potent inhibition of IDH1 mutants R132H, R132C, R132S, and R132L, respectively (Lin et al., 2019). Molecular docking results (Figure 4F) (PDB ID: 6U4J) demonstrate that the cyano moiety forms a hydrogen bond with the backbone amide NH of Leu120, while the linker benzylamine (NH) forms an H-bond with the carbonyl of Ile128. The 2-methoxy group of the benzonitrile ring sits is well located in a small hydrophobic cleft formed by Trp124 and Ile113. Both the carbonyl and the NH of the quinolinone form hydrogen bonds with Arg109 and Asp279, respectively. The 6-chloro substituent of the quinolinone ring fills in a hydrophobic pocket formed by residues Trp267, Ala258, Ile130, and Ile128. Compound **6** also exhibits excellent cell permeability, oral bioavailability, and ADME/PK properties.

After structural modification through compound **6**, FT-2102 was designed and synthesized. FT-2102 is an orally active inhibitor developed by Forma Therapeutics that can penetrate the blood-brain barrier (Caravella et al., 2020). The IC_50_ values of FT-2102 for mIDH1 R132H/R132C were 21.2 nM and 114 nM, respectively. FT-2102 can be used to treat AML and myelodysplastic syndromes [NCT02719574] (Megías-Vericat et al., 2019). Phase II trials of the drug in glioma and glioblastoma multiforme are underway [NCT03684811]. The recent results show that patients treated with the drug increase the expression of markers of hemoglobinization and erythroid differentiation, reduce leukemic stem cell populations, and potentiate apoptosis.

### Bis-imidazole phenol

Deng et al. (2015) discovered a class of bisimidazole phenols (VVS) as mIDH1 inhibitors through high-throughput screening (Figure 4G). Crystallographic studies show that VVS binds to the dimer interface in an allosteric manner and directly contacts Asp279 at the metal ion binding site, competitively inhibiting the binding of Mg^2+^ to protein, but not substrates and NADPH cofactors inhibition (Figure 4H) (PDB ID: 6UMX). VVS inhibits protease activity with IC_50_ of 0.01 μM. These results show that targeting divalent cation binding residue can selectively inhibit the activity of mIDH1. This result suggests that differences in magnesium binding between wild-type and mutant enzymes may contribute to the selectivity of inhibitors for the mutant enzyme.

### Tetrahydro pyrazolopyridine

GSK321 is a highly potent mIDH1 inhibitor with IC_50_ of 4.6 nM for mIDH1 R132H, which was developed by GlaxoSmithKline after optimizing the structure of GSK990 (Figure 4I) (Okoye-Okafor et al., 2015). GSK321 binds to the allosteric pocket when the human IDH1 enzyme is in an open state (Figure 4J) (PDB ID: 5DE1). In the GSK321-mIDH1 complex, the inhibitor does not make contact with NADP + or the mutated residue at His132. GSK321 binds to each of the pocket of IDH1 R132H monomers lined on three sides by Ile128, Pro127, Trp124, Arg119, and Leu120. GSK321 can induce myeloid differentiation in IDH1 mutant cells at the level of leukemic blasts and more stem cells but is still in preclinical studies.

### DS-1001b

**FIGURE 5:**
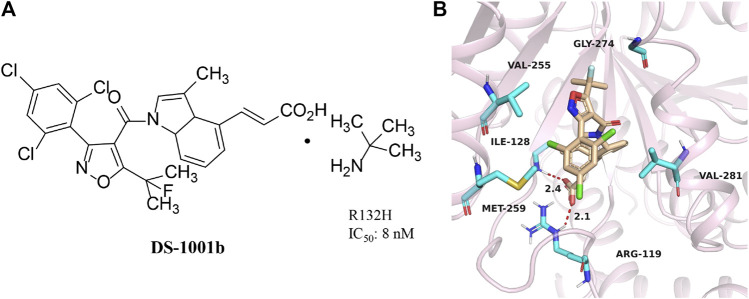
The structure of DS-1001b **(A)** and binding mode with mIDH1 **(B)**.

DS-1001b is a mIDH1 inhibitor reported by Daiichi Sankyo for the treatment of chondrosarcoma (Figure 5A) (Machida et al., 2020). DS-1001b impairs the proliferation of chondrosarcoma cells with IDH1 mutations *in vitro* and *vivo* (Nakagawa et al., 2019). DS-1001b reverses epigenetic changes caused by abnormal histone modifications. Various data suggest that DS-1001b may be an effective drug for the treatment of chondrosarcoma. The effect of this drug on recurrent or progressive gliomas is currently being studied clinically [NCT03030066]. Molecular docking displays that IDH1 forms a dimer (Figure 5B) (PDB ID:6IO0). DS-1001b binds to the allosteric pocket located in the dimer surface and stabilize the conformation of IDH1 R132C to the “open” inactive form. This conformational change disrupts the spatial arrangement of Asp residues (Asp 275, Asp 279, and Asp 252 in another protomer) that form the binding site of a catalytically important divalent cation. This conformational change also reduces the affinity for the substrate α-KG, since coordinate bond formed with the divalent cation is necessary for α-KG binding. As a result, DS-1001b inhibits the overall catalytic activity of mIDH1 by lowering the binding affinity of both divalent cation and substrate.

### 3-aryl-4-indolyl-maleimides

**FIGURE 6:**
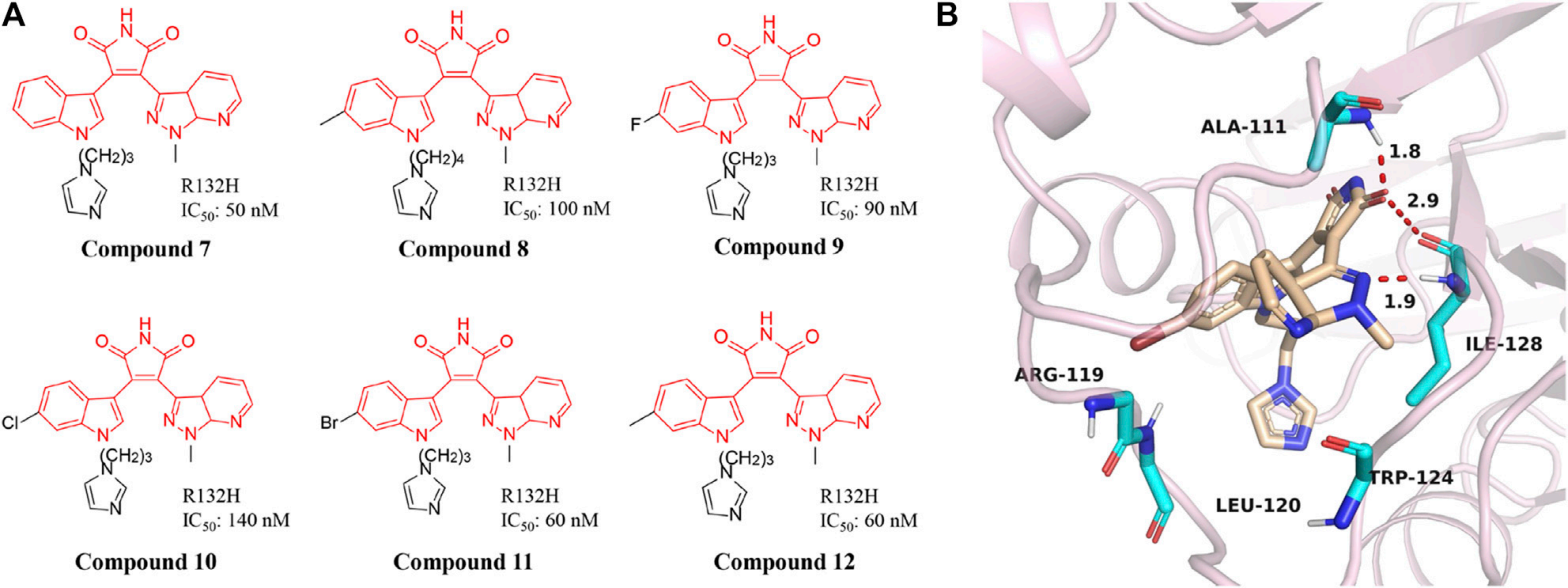
The structure of compound 7–12 **(A)** and binding mode with mIDH1 **(B)**.

A series of novel IDH1 R132H inhibitors were obtained through high-throughput screening and structure optimization (Compounds **7**–**12**) (Figure 6A) (Hu et al., 2018; Liu et al., 2019). Most of the compounds in this series show a high inhibitory effect. Molecular docking results describe that NH and carbonyl group in the maleimide ring of compounds can form tight interaction with the Ile128 and Ala111 of IDH1/R132H *via* the hydrogen-bonding network (Figure 6B) (PDB ID: 5DE1). The 7-position nitrogen of the 7-azaindazole ring forms another H-bond with Arg119. Both 7-azaindazole and indole ring of compound 7–12 have π-π interaction (T-shaped) with Trp124 residue. In addition, bromine at the 6-position of the indole ring can fit into the hydrophobic pocket composed of Val255 and Met259. The distances from the 3-position nitrogen of the imidazole ring to the NH of Arg119 and Leu120 are 4.29 Å and 3.47 Å, respectively, which makes it possible for the N atom to have water-mediated interaction with Arg119 and Leu120. It can effectively inhibit the production of 2-HG in U87MG cells. In addition, it reverses the differentiation block of myeloid leukemia cell lines, providing a basis for future studies.

### 1-hydroxy-pyridin-2-one

**FIGURE 7:**
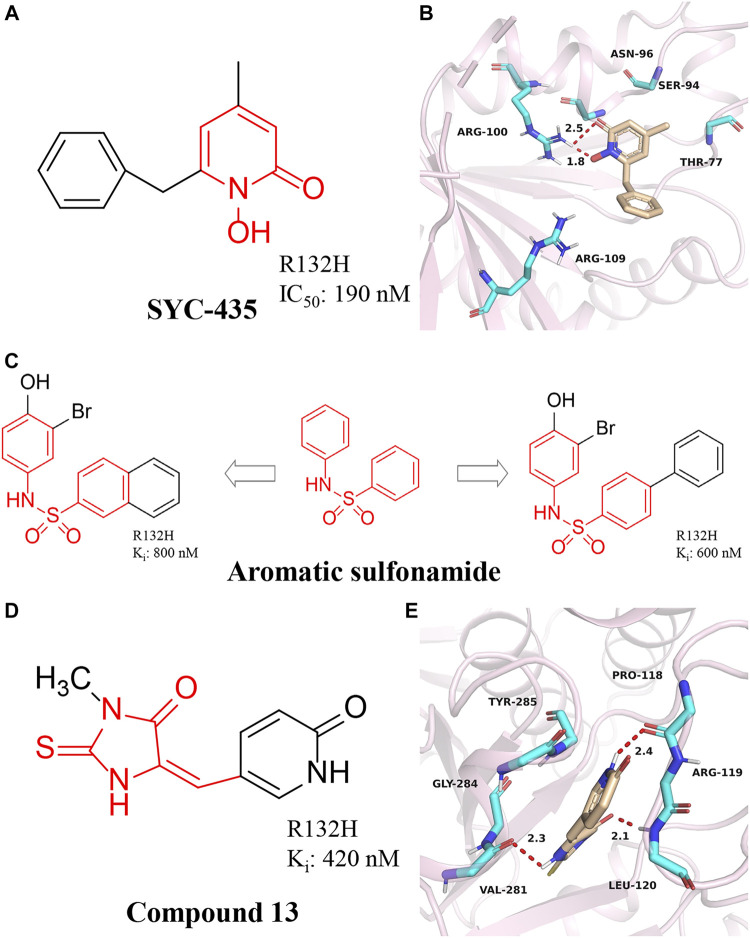
**(A,B)** The structure of SYC-435 and binding mode with mIDH1. **(C)** The structure of aromatic sulfonamide compounds. **(D,E)** The structure of Compound 13 and binding mode with mIDH1.

A series of 1-hydroxy-pyridin-2-ones were synthesized by Liu et al. (2014) (Zheng et al., 2013). The most representative compound is SYC-435 (Figure 7A). Liu et al. (2014) found that SYC-435 has K_i_ values as low as 140 nM. Notably, SYC-435 has BBB permeability, which may facilitate the development of novel mIDH1 inhibitors to treat gliomas. Molecular docking results show that SYC-435 is surrounded by pockets formed by Arg100, Ser94, Thr77, Asn96, Arg109, and NADPH (Figure 7B) (PDB ID: 4I3L). The two oxygen atoms in the compound form hydrogen bonds with the two nitrogen atoms of Arg100. In addition, the 4-position methyl group can be embedded into a hydrophobic pocket, which can also greatly enhance the efficacy of the compound.

### Aromatic sulfonamide compounds

A series of aromatic sulfonamide compounds were found to be novel and potent inhibitors of mIDH1 R132H (Figure 7C) (Wu et al., 2018). These novel compounds have K_i_ values as low as 0.6 μM. Importantly, these compounds exhibit equally satisfactory anti-proliferative activity against BT142 glioma cells with the R132H IDH1 mutation. Meanwhile, aromatic sulfonamide compounds did not affect the growth of BXD-3752 glioma cells without IDH1 mutation, which indicates that aromatic sulfonamide compounds will have a higher selectivity for IDH1 mutated glioma cells. These results suggest further characterization and optimization of these compounds are warranted to find a clinically useful drug targeting IDH1 mutated gliomas.

### 2-thiohydantoin compounds

Wu et al. (2015) reported the synthesis, structure-activity relationship, enzyme kinetics, and binding thermodynamic studies of a series of novel 2-thiohydantoins and related compounds. Compound **13** (Figure 7D) is located deep within the cleft between the two protein homodimers (Figure 7E) (PDB ID: 4XS3), sitting in a pocket surrounded by Thr77, Ser94, Asn96, Gly97, Arg100, Asn101, Arg109, and NADPH residues. The nearly flat inhibitors are located on a “bed surface” composed of Gly97, Asn96, and Ser94, with the distance between the two nearly parallel planes being about 3.3–3.6 Å. Two partially negatively charged O atoms form three H bonds and electrostatically interact with the positively charged side chains of Arg100 and Asn101. The S atom of compound **13** has good primary hydrophobic interactions with Thr77 and NADPH. Compound **13** is a potent inhibitor of mIDH1 with K_i_ as low as 420 nM. Compound **13** reduces the cellular concentration of D-2-HG, decreases the levels of histone methylation, and inhibits the proliferation of BT142 gliomas cells with mIDH1 R132H.

### 8-membered ring sulfonamides

**FIGURE 8:**
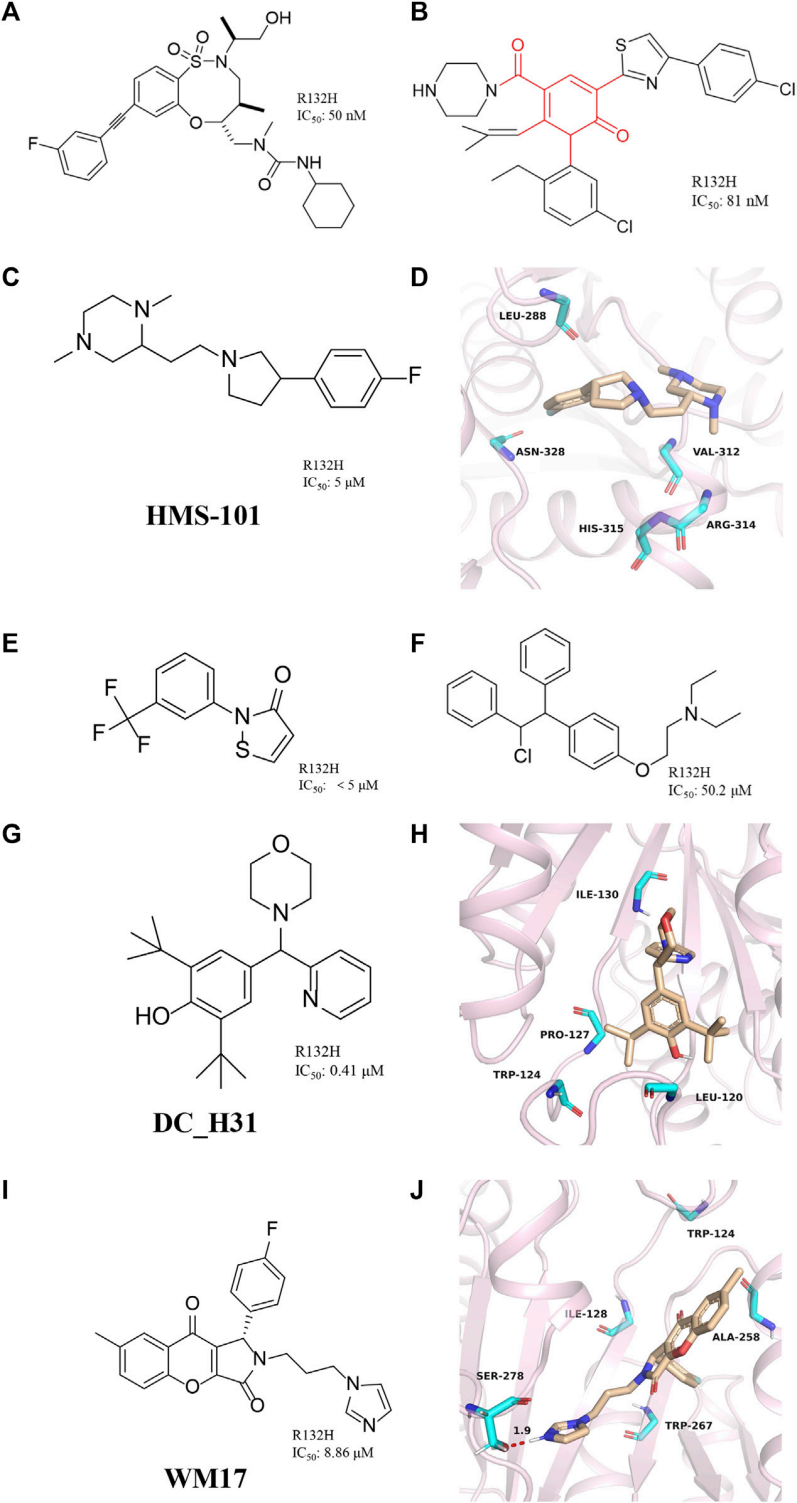
**(A)** The structure of BRD2879. **(B)** The structure of compound 14. **(C,D)** The structure of HMS-101and binding mode with mIDH1. **(E)** The structure of 2-(3-(trifluoromethyl) phenyl) isothiazol-3(2H)-one. **(F)** The structure of Clomifene citrate. **(G,H)** The structure of DC_H31 and binding mode with mIDH1. **(I,J)** The structure of WM17 and binding mode with mIDH1.

The sulfonamide BRD2879 (Figure 8A) with an eight-membered ring can also inhibit the production of 2-HG *in vivo* without obvious toxicity, but its low solubility and special pharmacokinetic properties prevent its use *in vivo* (Law et al., 2016). Although the current potency of BRD2879 is not obvious enough, the structure-activity relationship of the compound has been revealed through the exploration of SAR, and its solubility, selectivity, and metabolic sensitivity may be improved through structural modification. BRD2879 represents a new structural class of mIDH1 inhibitors that may be useful for studying mIDH1 inhibitor and its role in cancer after optimization.

### 2H-1λ2-Pyridin-2-one

Jason M. *et al.* reported a class of mIDH1 inhibitors with 2H-1λ2-Pyridin-2-one backbone (Rohde et al., 2021). The most potent compound **14** (Figure 8B) inhibits IDH1 R132H/R132C with IC_50_ values of 81 and 72 nM, respectively. Compound 14 is stronger than AG-120 in inhibiting the effect of 2-HG. In a PK/PD model, compound 14 shows sustained tumor concentrations (7 μM after 48 h) after a single oral dose of 30 mg/kg, and at later time points (16–48 h) the levels of 2-HG are reduced more than the approved mIDH1 drug AG-120.

### 2-[2-[3-(4-fluorophenyl) pyrrolidin-1-yl] ethyl]-1,4-dimethylpiperazine

Anuhar *et al.* obtained a series of mIDH1 inhibitors with 2-[2-[3-(4-fluorophenyl) pyrrolidin-1-yl] ethyl]-1,4-dimethylpiperazine skeleton by computational screening (Figure 8C), which was named HMS-101 (Chaturvedi, et al., 2020). The crystal structure shows that HMS-101 binds to the binding pocket of mIDH1 (Figure 8D) (PDB ID: 6Q6F). The piperazine ring of HMS-101 forms a good interaction with the main chain nitrogen of Val312, the pyrrolidine ring, and the side chain of Arg314. In addition, a halogen bond between the fluorophenyl and Asn328 is found, and several hydrophobic interactions with residues Leu288, Gly310, and Val312 stabilize the binding. The experimental study found that the inhibitory effect of HMS101 on mIDH1 enzyme activity was dose-dependent, and the IC_50_ value was 4 µM. It was found that HMS-101 could significantly induce apoptosis in mIDH1 AML cells and mouse CD341 myeloid cells (Chaturvedi et al., 2013).

### 2-(3-(trifluoromethyl) phenyl) isothiazol-3(2H)-one

A newly mIDH1 inhibitor named 2-(3-(trifluoromethyl) phenyl) isothiazol-3(2H)-one was reported (Figure 8E) (Kim et al., 2015). 2-(3-(trifluoromethyl) phenyl) isothiazol-3(2H)-one does not interfere with the IDH1 activity, demonstrating the selectivity of this compound against mIDH1 R132H. It has also been shown to be effective in inhibiting the production of D-2HG. Unfortunately, it has not been pointed out how this molecule selectively inhibits mIDH1 activity.

### Clomifene citrate

The candidate drugs of the drug library are reused by the method of virtual screening (Ma et al., 2013). Clomifene is a good example of an application (Figure 8F), which is used as a modifier for the treatment of female infertility (Dickey and Holtkamp, 1996; Hughes et al., 1996; Tobinick, 2009). It was then proposed as a testosterone level reversal agent in men (Jungheim and Odibo, 2010). Clomifene is now identified as a mIDH1 inhibitor using virtual screening (Kattih et al., 2021). Clomifene has been shown to reduce the production of D-2HG *in vivo* and *vitro*. At the same time, it can reduce the level of H3K9me3 in tumor tissue. Such finding could allow Clomifene to be investigated as a lead compound to utilize Clomifene’s backbone to synthesize more efficient mIDH1 inhibitors.

### DC_31

A team from Huazhong Agricultural University obtained DC_H31 as a mIDH1 inhibitor through high-throughput screening (Figure 8G) (Duan, et al., 2019). Molecular docking result shows that DC_H31 does not directly act on Tyr139, but occupies an allosteric site between the two monomers of IDH1-R132H (Figure 8H) (PDB ID: 5LGE). DC_H31 can form two hydrogen bonds with the carbonyl group of Gln277 and the hydroxyl group of Ser280 in mIDH1 R132H, respectively. In addition, the pyridine ring on the DC_H31 molecule can form three edge-to-face forces with Trp124, Tyr285, and Trp267 residues in three different directions, which cooperate with the highly hydrophobic surrounding the allosteric pocket. This environment helps stabilize the DC_H31 confirmation within the pocket to exert inhibitory activity. Preliminary studies at the cellular level showed that DC_H31 inhibits the enzyme activity of mIDH1 in HT1080 cells, thereby reducing the production of intracellular 2-HG and down-regulating the transcription level of the downstream gene SOX2, as well as promoting the transcription and expression of the downstream gene GFAP. But its mechanism of action still needs to be further explored to develop inhibitors with better activity.

### WM17

Zhang, et al. (2021) described a novel mIDH1 inhibitor, WM17 (Figure 8I), which was obtained by virtual and enzymatic screening. WM17 can form hydrophobic interactions and hydrogen bonding interactions by binding to the allosteric tetrahelical center of IDH1-R132H consisting of amino acids TRP124, ILE128, ALA258, and TRP267 (Figure 8J) (PDB ID: 4UMX). It can bind and increase the thermostability of mIDH1 protein in endogenous hybrid cells and exogenous overexpressing cells. WM17 inhibits cell migration and reverses the accumulation of D-2-HG and histone hypermethylation in IDH1 mutant cells. Further study warrants the development of WM17 as a therapeutic agent for IDH1-mutant gliomas.

### CRUK-MI

**FIGURE 9:**
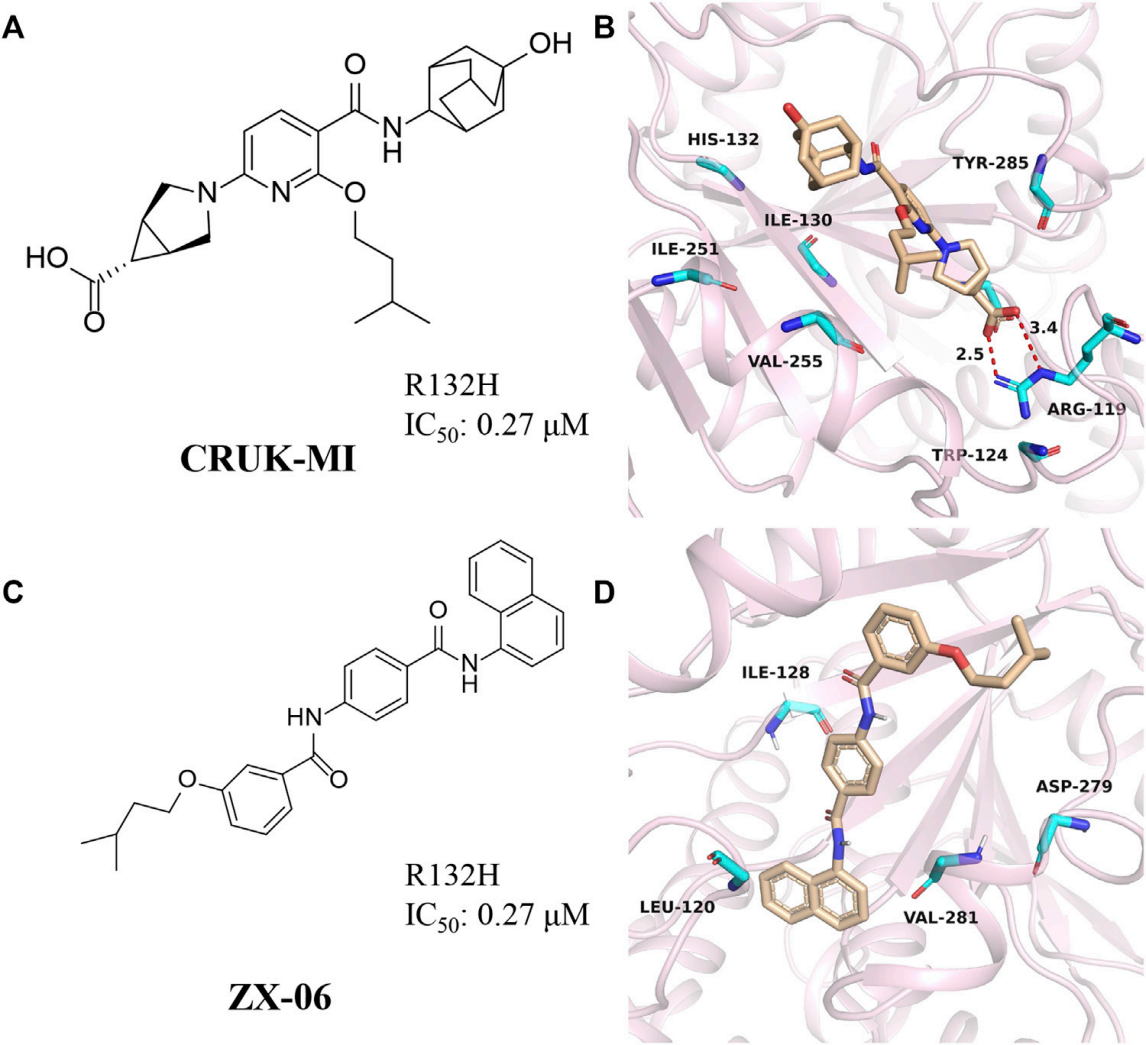
**(A,B)** The structure of CRUK-MI and binding mode with mIDH1. **(C,D)** The structure of ZX-06 and binding mode with mIDH1.

CRUK-MI was developed by AstraZeneca and Manchester Institute of Technology Cancer Institute (Figure 9A) (Jones et al., 2016), with IC_50_ values of 0.27 μM and 0.47 μM for mIDH1 R132H and R132C, respectively. CRUK-MI can bind to the interface of the two components of the protein dimer. The ionic interaction between the carboxylic acid of the compound and Arg119 forms a hydrogen bond (Figure 9B) (PDB ID: 5L58), aromatic stacking between the pyridyl and Tyr285, lipophilic contacts between the isopentyl moiety and the enclosed dimeric pocket around Val255 and Met 259 and looser lipophilic contacts around the adamantyl moiety. When tested using *in vitro* assays, the most encouraging compound promotes the differentiation of AML cells in patient-derived with IDH1 R132H, but not in IDH1 wild-type AML cells, confirming on-target activity in the primary cell setting.

### ZX06

Cross docking-based virtual screening identified ZX06 as a mIDH1 inhibitor that exhibited potent inhibitory activity and high selectivity against WT-IDH1 (Figure 9C) (Zou et al., 2018). During molecular docking (Figure 9D) (PDB ID: 4UMX), if ZX06 is docked into the Seg-2 pocket, the 3-nitrogen of the pyridine ring of ZX06 can form a hydrogen bond with Ile128. The amide linker forms two strong hydrogen bonds with Leu120 and Val281 with bond lengths of 2.1 Å and 1.8 Å, respectively. If docked into the Mg^2+^ pocket, the NH of the amide group of ZX06 forms a hydrogen bond with Asp279, and the molecule is tightly located in the divalent magnesium pocket, similar to VVS. It is worth noting that ZX06 has a good ability to penetrate the blood-brain barrier, which provides good support for the further development of mIDH1 inhibitors. The compound holds promise as a lead compound for the treatment of patients with IDH1-mutated brain cancer.

### AQ-714/41674992

**FIGURE 10:**
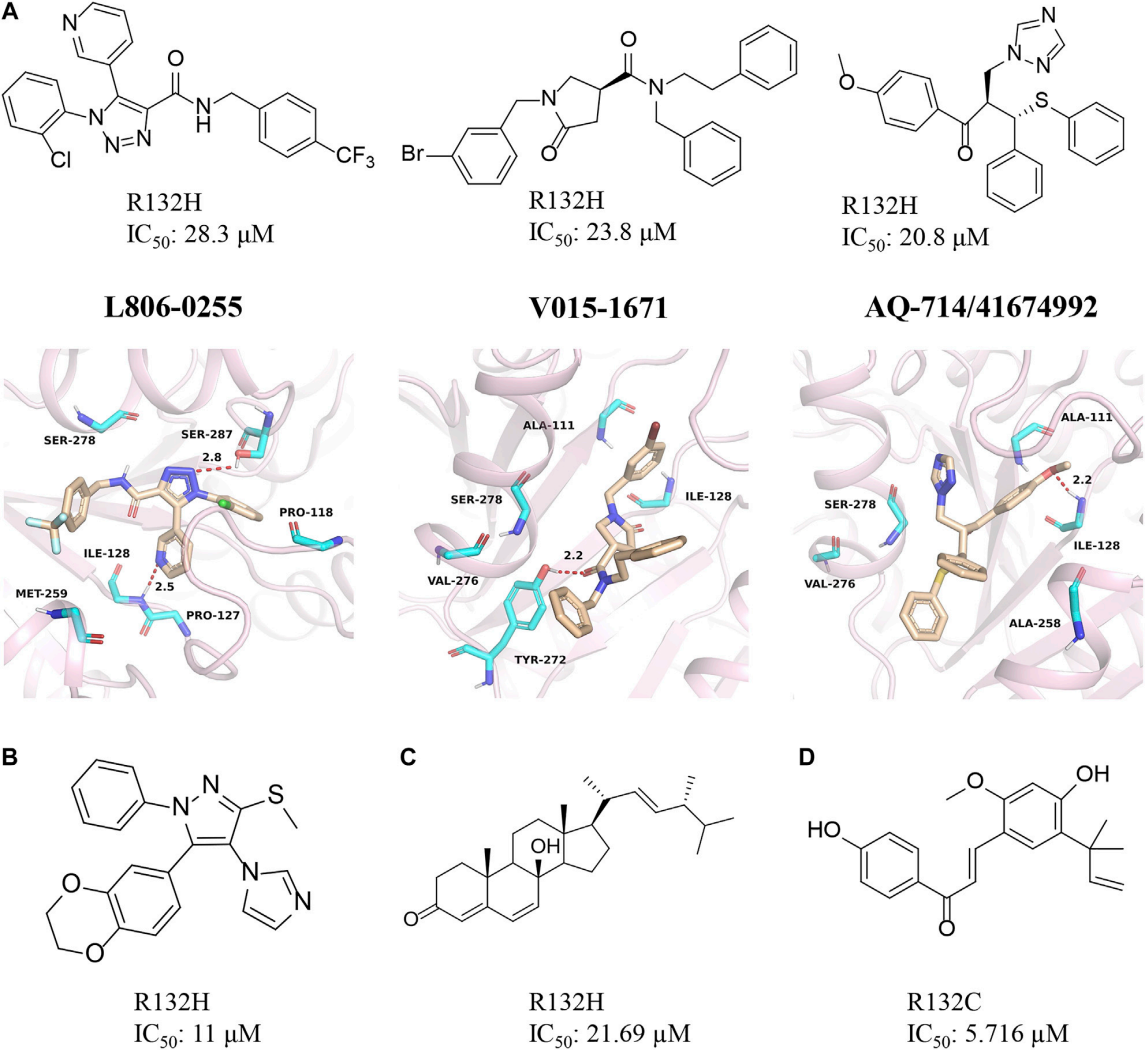
**(A)** The structure of L806-0255, V015-1671, AQ-714/41674992, and binding mode with mIDH1. **(B)** The structure of KRC-09. **(C)** The structure of Steroids. **(D)** The structure of licochalone **(A)**

Our group obtained three novel structures that are expected to be IDH1 inhibitors through virtual screening based on comparative structures, namely L806-0255, V015-1671, and AQ-714/41674992 (Figure 10A) (Wang et al., 2020). The enzyme activity test found that AQ-714/41674992 has the highest inhibition efficiency. Molecular docking results reveal that these three molecules can dock to the binding pocket consisting of hydrophobic residues (PDB ID: 5TQH, and 6B0Z), and the compounds form intermolecular hydrogen bonds with key residues, thereby stabilizing the complex. Among them, L806-0255 and V015-1671 form key hydrogen bonds with ILE128, while V015-1671 and AQ-714/41674992 form key hydrogen bonds with ALA111. Moreover, the hydrophobic contacts formed between compounds with surrounding residues, such as VAL276, SER278, SER287, ILE128, and PRO118 also contribute to enhanced binding of the small molecule inhibitor to mIDH1 R132H. AQ-714/41674992 is expected to become a novel IDH1 inhibitor through further structural optimization.

### KRC

Kang et al. (2020) discovered a compound named KRC-09 through high-throughput screening (Figure 10B). This is a completely new compound that has not been reported before and the IC_50_ value of KRC-09 in the enzymatic activity assay is 11 µM. In an experiment that inhibits the production of 2-HG, KRC-09 exhibits stronger anti-IDH1 activity than AGI-5198. The compound may become a new potent inhibitor after structural optimization.

### Steroid

The monomeric compounds separated and purified from traditional Chinese medicine may also have inhibitory activity against mIDH1. Deng *et al.* screened the active ingredients of Ganoderma lucidum and obtained a natural sterol compound (Figure 10C) (Zheng M et al., 2018). The computational results show that the steroid has a very high affinity for mIDH1. Enzyme kinetic studies indicate that this substance inhibits mIDH1 activity in a non-competitive manner. The steroid can effectively inhibit the production of D-2HG in HT-1080 cells with IC_50_ of 35.97 µM. In addition, the steroid also reduces the methylation level of histone H3K9me3 in HT-1080 cells. Overall, this is a very potent inhibitor for mIDH1.

### Licochalcone A

The enzyme activity test found that licochalone A also had inhibitory activity against mIDH1 (Figure 10D) (Hu et al., 2020). The IC_50_ of licochalone A for the inhibitory activity against mIDH1 R132C is 5.176 µM. Molecular docking revealed that this molecule may occupy the allosteric pocket between the two monomers of the IDH1 homodimer. But it is worth noting that this compound does not bind well to the mIDH1 R132H. It is also necessary to use licochalone A as a starting point for the development of mIDH1 inhibitor.

## Perspective

In recent years, there have been numerous small molecule inhibitors targeting mIDH1, and several drugs have entered clinical studies, but only AG-120 has been approved by the FDA for marketing. Most of the discovered small molecule compounds still have problems such as insufficient *in vitro* activity and poor druggability. The inhibitors that have entered clinical studies have reached nanomolar levels of *in vitro* activity but still suffer from poor cellular activity, poor selectivity, or poor pharmacokinetic properties. These factors have greatly limited the study of the molecular mechanisms of mIDH1 *in vitro* and *vivo* and the development of its inhibitors.

At present, the discovery of mIDH1 inhibitors is mainly carried out by high-throughput screening (Pirozzi et al., 2013). Although this technique has the characteristics of rapid, sensitive detection and accuracy, it is still limited by the size of compounds in the database. With the development of computer technology, virtual screening has been widely used in the drug discovery process. Screening the compound databases through various computational methods to find small molecules that bind better to mIDH1, and then obtaining more effective small molecule inhibitors through structural optimization will help improve the efficiency of drug discovery (Wang et al., 2021). Particularly, natural products or traditional Chinese medicine monomers can also be considered important sources of mIDH1 inhibitors during the process of virtual screening based on the virtual database. The available studies have shown that screening of Chinese medicine monomers as mIDH1 inhibitors is a feasible route. In addition, the current popular research on IDH mutation therapy also includes IDH mutation immunotherapy (Schumacher et al., 2014; Pellegatta et al., 2015) and IDH mutation enzyme reset (Reitman et al., 2012). With the development of the global pharmaceutical industry, it is believed that diseases caused by IDH1 mutations will be cured shortly.

## References

[B1] Abou-Alfa G. K. Macarulla T. Javle M. M. Kelley R. K. Lubner S. J. Adeva J. (2020). Ivosidenib in IDH1-mutant, chemotherapy-refractory cholangiocarcinoma (ClarIDHy): a multicentre, randomised, double-blind, placebo-controlled, phase 3 study. Lancet. Oncol. 21 (6), 796–807. 10.1016/s1470-2045(20)30157-1 32416072 PMC7523268

[B2] Azzi G. Velez M. Mathias-Machado M. C. (2014). Isocitrate dehydrogenase mutations in chondrosarcoma: the crossroads between cellular metabolism and oncogenesis. Curr. Opin. Oncol. 26 (4), 403–407. 10.1097/cco.0000000000000092 24867810

[B3] Caferro T. R. Cho Y. S. Costales A. Q. Lei H. Lenoir F. Levell J. R. (2015). 3-pyrimidin-4-yl-oxazolidin-2-ones as inhibitors of mutant IDH. US.

[B4] Cao H. Zhu G. Sun L. Chen G. Ma X. Luo X. (2019). Discovery of new small molecule inhibitors targeting isocitrate dehydrogenase 1 (IDH1) with blood-brain barrier penetration. Eur. J. Med. Chem. 183, 111694. 10.1016/j.ejmech.2019.111694 31561044

[B5] Caravella J. A. Lin J. Diebold R. B. Campbell A. M. Ericsson A. Gustafson G. (2020). Structure-based design and identification of FT-2102 (olutasidenib), a potent mutant-selective IDH1 inhibitor. J. Med. Chem. 63 (4), 1612–1623. 10.1021/acs.jmedchem.9b01423 31971798

[B6] Chaturvedi A. Araujo Cruz M. M. Jyotsana N. Sharma A. Yun H. Görlich K. (2013). Mutant IDH1 promotes leukemogenesis *in vivo* and can be specifically targeted in human AML. Blood 122 (16), 2877–2887. 10.1182/blood-2013-03-491571 23954893

[B7] Chaturvedi A. Goparaju R. Gupta C. Weder J. Klünemann T. Araujo Cruz M. M. (2020). *In vivo* efficacy of mutant IDH1 inhibitor HMS-101 and structural resolution of distinct binding site. Leukemia 34 (2), 416–426. 10.1038/s41375-019-0582-x 31586149 PMC6995692

[B8] Chaturvedi A. Herbst L. Pusch S. Klett L. Goparaju R. Stichel D. (2017). Pan-mutant-IDH1 inhibitor BAY1436032 is highly effective against human IDH1 mutant acute myeloid leukemia *in vivo* . Leukemia 31 (10), 2020–2028. 10.1038/leu.2017.46 28232670 PMC5629366

[B9] Chen R. D. Gadal P. (1990). Structure, functions and regulation of NAD and NADP dependent isocitrate dehydrogenases in higher plants and in other organisms. Plant Physiol. Biochem. 28 (3), 411–427.

[B10] Chen R. Smith-Cohn M. Cohen A. L. Colman H. (2017). Glioma subclassifications and their clinical significance. Neurotherapeutics 14 (2), 284–297. 10.1007/s13311-017-0519-x 28281173 PMC5398991

[B11] Chen Y. Shao X. Zhao X. Ji Y. Liu X. Li P. (2021). Targeting protein arginine methyltransferase 5 in cancers: Roles, inhibitors and mechanisms. Biomed. Pharmacother. 144, 112252. 10.1016/j.biopha.2021.112252 34619493

[B12] Cho Y. S. Levell J. R. Liu G. Caferro T. Sutton J. Shafer C. M. (2017). Discovery and evaluation of clinical candidate IDH305, a brain penetrant mutant IDH1 inhibitor. ACS Med. Chem. Lett. 8 (10), 1116–1121. 10.1021/acsmedchemlett.7b00342 29057061 PMC5641959

[B13] Chowdhury R. Yeoh K. K. Tian Y. M. Hillringhaus L. Bagg E. A. Rose N. R. (2011). The oncometabolite 2-hydroxyglutarate inhibits histone lysine demethylases. EMBO Rep. 12 (5), 463–469. 10.1038/embor.2011.43 21460794 PMC3090014

[B14] Dang L. Su S. M. (2017). Isocitrate dehydrogenase mutation and (R)-2-Hydroxyglutarate: From basic discovery to therapeutics development. Annu. Rev. Biochem. 86, 305–331. 10.1146/annurev-biochem-061516-044732 28375741

[B15] Davis M. I. Gross S. Shen M. Straley K. S. Pragani R. Lea W. A. (2014). Biochemical, cellular, and biophysical characterization of a potent inhibitor of mutant isocitrate dehydrogenase IDH1. J. Biol. Chem. 289 (20), 13717–13725. 10.1074/jbc.M113.511030 24668804 PMC4022846

[B16] Davis M. Pragani R. Popovici-Muller J. Gross S. Thorne N. Salituro F. (2010). “ML309: A potent inhibitor of R132H mutant IDH1 capable of reducing 2-hydroxyglutarate production in U87 MG glioblastoma cells,” in Probe reports from the NIH molecular libraries program (Bethesda (MD): National Center for Biotechnology Information US. 23905201

[B17] Deng G. Shen J. Yin M. McManus J. Mathieu M. Gee P. (2015). Selective inhibition of mutant isocitrate dehydrogenase 1 (IDH1) via disruption of a metal binding network by an allosteric small molecule. J. Biol. Chem. 290 (2), 762–774. 10.1074/jbc.M114.608497 25391653 PMC4294499

[B18] Dickey R. P. Holtkamp D. E. (1996). Development, pharmacology and clinical experience with clomiphene citrate. Hum. Reprod. Update 2 (6), 483–506. 10.1093/humupd/2.6.483 9111183

[B19] Döhner H. Wei A. H. Löwenberg B. (2021). Towards precision medicine for AML. Nat. Rev. Clin. Oncol. 18 (9), 577–590. 10.1038/s41571-021-00509-w 34006997

[B20] Dowdy T. Zhang L. Celiku O. Movva S. Lita A. Ruiz-Rodado V. (2020). Sphingolipid pathway as a source of vulnerability in IDH1^ *mut* ^ glioma. Cancers (Basel) 12 (10), E2910. 10.3390/cancers12102910 33050528 PMC7601159

[B21] Duan Z. Liu J. Niu L. Wang J. Feng M. Chen H. (2019). Discovery of DC_H31 as potential mutant IDH1 inhibitor through NADPH-based high throughput screening. Bioorg. Med. Chem. 27 (15), 3229–3236. 10.1016/j.bmc.2019.05.040 31208797

[B22] Duchmann M. Micol J. B. Duployez N. Raffoux E. Thomas X. Marolleau J. P. (2021). Prognostic significance of concurrent gene mutations in intensively treated patients with IDH-mutated AML: an ALFA study. Blood 137 (20), 2827–2837. 10.1182/blood.2020010165 33881523

[B23] Fan B. Dai D. DiNardo C. D. Stein E. de Botton S. Attar E. C. (2020a). Clinical pharmacokinetics and pharmacodynamics of ivosidenib in patients with advanced hematologic malignancies with an IDH1 mutation. Cancer Chemother. Pharmacol. 85 (5), 959–968. 10.1007/s00280-020-04064-6 32296873

[B24] Fan B. Mellinghoff I. K. Wen P. Y. Lowery M. A. Goyal L. Tap W. D. (2020b). Clinical pharmacokinetics and pharmacodynamics of ivosidenib, an oral, targeted inhibitor of mutant IDH1, in patients with advanced solid tumors. Investig. New Drugs 38 (2), 433–444. 10.1007/s10637-019-00771-x 31028664 PMC7066280

[B25] Fiorentini A. Capelli D. Saraceni F. Menotti D. Poloni A. Olivieri A. (2020). The time has come for targeted therapies for AML: Lights and shadows. Oncol. Ther. 8 (1), 13–32. 10.1007/s40487-019-00108-x 32700072 PMC7359996

[B26] Gerecke C. Schumacher F. Berndzen A. Homann T. Kleuser B. (2020). Vitamin C in combination with inhibition of mutant IDH1 synergistically activates TET enzymes and epigenetically modulates gene silencing in colon cancer cells. Epigenetics 15 (3), 307–322. 10.1080/15592294.2019.1666652 31505989 PMC7028341

[B27] Gokul S. Rajanikant G. K. (2017). Research highlights BAY 1436032: A novel pan-mutant IDH1 inhibitor extends survival of mice with experimental brain tumors. CNS Neurol. Disord. Drug Targets 16 (6), 636–637. 10.2174/1871527316999170505104203 28901861

[B28] Golub D. Iyengar N. Dogra S. Wong T. Bready D. Tang K. (2019). Mutant isocitrate dehydrogenase inhibitors as targeted cancer therapeutics. Front. Oncol. 9, 417. 10.3389/fonc.2019.00417 31165048 PMC6534082

[B29] Han S. Liu Y. Cai S. J. Qian M. Ding J. Larion M. (2020). IDH mutation in glioma: molecular mechanisms and potential therapeutic targets. Br. J. Cancer 122 (11), 1580–1589. 10.1038/s41416-020-0814-x 32291392 PMC7250901

[B30] Heuser M. Palmisiano N. Mantzaris I. Mims A. DiNardo C. Silverman L. R. (2020). Safety and efficacy of BAY1436032 in IDH1-mutant AML: phase I study results. Leukemia 34 (11), 2903–2913. 10.1038/s41375-020-0996-5 32733012 PMC7584476

[B31] Hu C. Zuo Y. Liu J. Xu H. Liao W. Dang Y. (2020). Licochalcone A suppresses the proliferation of sarcoma HT-1080 cells, as a selective R132C mutant IDH1 inhibitor. Bioorg. Med. Chem. Lett. 30 (2), 126825. 10.1016/j.bmcl.2019.126825 31836442

[B32] Hu Y. Gao A. Liao H. Zhang M. Xu G. Gao L. (2018). 3-(7-Azaindolyl)-4-indolylmaleimides as a novel class of mutant isocitrate dehydrogenase-1 inhibitors: Design, synthesis, and biological evaluation. Arch. Pharm. 351 (10), e1800039. 10.1002/ardp.201800039 30113716

[B33] Hughes E. Collins J. Vandekerckhove P. (1996). Withdrawn: Clomiphene citrate for ovulation induction in women with oligo-amenorrhoea. Cochrane Database Syst. Rev. 1, Cd000056. 10.1002/14651858.CD000056.pub2 PMC10866097 17636579

[B34] Hurley J. H. Thorsness P. E. Ramalingam V. Helmers N. H. Koshland D. E. Jr. Stroud R. M. (1989). Structure of a bacterial enzyme regulated by phosphorylation, isocitrate dehydrogenase. Proc. Natl. Acad. Sci. U. S. A. 86 (22), 8635–8639. 10.1073/pnas.86.22.8635 2682654 PMC298342

[B35] Imabayashi F. Aich S. Prasad L. Delbaere L. T. (2006). Substrate-free structure of a monomeric NADP isocitrate dehydrogenase: an open conformation phylogenetic relationship of isocitrate dehydrogenase. Proteins 63 (1), 100–112. 10.1002/prot.20867 16416443

[B36] Jia P. Wu Y. Du H. Yang L. Zhang Z. Ma T. (2019). I-8, a novel inhibitor of mutant IDH1, inhibits cancer progression *in vitro* and *in vivo* . Eur. J. Pharm. Sci. 140, 105072. 10.1016/j.ejps.2019.105072 31518680

[B37] Jiang B. Zhao W. Shi M. Zhang J. Chen A. Ma H. (2018). IDH1 R132 mutant promotes tumor formation through downregulating p53. J. Biol. Chem. 293, 001385. 10.1074/jbc.RA117.001385 PMC6016470 29743236

[B38] Jingtao L. Meiyu C. Haiying H. Wei Q. Ri Z. Xuzhang L. (2021). Additional mutations in IDH1/2-mutated patients with acute myeloid leukemia. Int. J. Lab. Hematol. 43 (6), 1483–1490. 10.1111/ijlh.13648 34270876

[B39] Jones S. Ahmet J. Ayton K. Ball M. Cockerill M. Fairweather E. (2016). Discovery and optimization of allosteric inhibitors of mutant isocitrate dehydrogenase 1 (R132H IDH1) displaying activity in human acute myeloid leukemia cells. J. Med. Chem. 59 (24), 11120–11137. 10.1021/acs.jmedchem.6b01320 28002956

[B40] Jungheim E. S. Odibo A. O. (2010). Fertility treatment in women with polycystic ovary syndrome: a decision analysis of different oral ovulation induction agents. Fertil. Steril. 94 (7), 2659–2664. 10.1016/j.fertnstert.2010.03.077 20451181 PMC2953591

[B41] Kang C. H. Choi S. U. Son Y. H. Lee H. K. Jeong H. G. Yun C. S. (2020). Discovery of a novel chemical scaffold against mutant isocitrate dehydrogenase 1 (IDH1). Anticancer Res. 40 (9), 4929–4935. 10.21873/anticanres.14496 32878781

[B42] Kattih B. Shirvani A. Klement P. Garrido A. M. Gabdoulline R. Liebich A. (2021). IDH1/2 mutations in acute myeloid leukemia patients and risk of coronary artery disease and cardiac dysfunction-a retrospective propensity score analysis. Leukemia 35 (5), 1301–1316. 10.1038/s41375-020-01043-x 32948843 PMC8102189

[B43] Kim H. J. Choi B. Y. Keum Y. S. (2015). Identification of a new selective chemical inhibitor of mutant isocitrate dehydrogenase-1. J. Cancer Prev. 20 (1), 78–83. 10.15430/jcp.2015.20.1.78 25853107 PMC4384718

[B44] Kim N. I. Noh M. G. Kim J. H. Won E. J. Lee Y. J. Hur Y. (2020). Frequency and prognostic value of IDH mutations in Korean patients with cholangiocarcinoma. Front. Oncol. 10, 1514. 10.3389/fonc.2020.01514 33014795 PMC7461833

[B45] Koivunen P. Lee S. Duncan C. G. Lopez G. Lu G. Ramkissoon S. (2012). Transformation by the (R)-enantiomer of 2-hydroxyglutarate linked to EGLN activation. Nature 483 (7390), 484–488. 10.1038/nature10898 22343896 PMC3656605

[B46] Konteatis Z. Artin E. Nicolay B. Straley K. Padyana A. K. Jin L. (2020). Vorasidenib (AG-881): A first-in-class, brain-penetrant dual inhibitor of mutant IDH1 and 2 for treatment of glioma. ACS Med. Chem. Lett. 11 (2), 101–107. 10.1021/acsmedchemlett.9b00509 32071674 PMC7025383

[B47] Law J. M. Stark S. C. Liu K. Liang N. E. Hussain M. M. Leiendecker M. (2016). Discovery of 8-membered ring sulfonamides as inhibitors of oncogenic mutant isocitrate dehydrogenase 1. ACS Med. Chem. Lett. 7 (10), 944–949. 10.1021/acsmedchemlett.6b00264 27774134 PMC5066158

[B48] Levell J. R. Caferro T. Chenail G. Dix I. Dooley J. Firestone B. (2017). Optimization of 3-Pyrimidin-4-yl-oxazolidin-2-ones as allosteric and mutant specific inhibitors of IDH1. ACS Med. Chem. Lett. 8 (2), 151–156. 10.1021/acsmedchemlett.6b00334 28197303 PMC5304300

[B49] Lin J. Lu W. Caravella J. A. Campbell A. M. Diebold R. B. Ericsson A. (2019). Discovery and optimization of quinolinone derivatives as potent, selective, and orally bioavailable mutant isocitrate dehydrogenase 1 (mIDH1) inhibitors. J. Med. Chem. 62 (14), 6575–6596. 10.1021/acs.jmedchem.9b00362 31199148

[B50] Liu S. Abboud M. I. John T. Mikhailov V. Hvinden I. Walsby-Tickle J. (2021). Roles of metal ions in the selective inhibition of oncogenic variants of isocitrate dehydrogenase 1. Commun. Biol. 4 (1), 1243. 10.1038/s42003-021-02743-5 34725432 PMC8560763

[B51] Liu X. Hu Y. Gao A. Xu M. Gao L. Xu L. (2019). Synthesis and biological evaluation of 3-aryl-4-indolyl-maleimides as potent mutant isocitrate dehydrogenase-1 inhibitors. Bioorg. Med. Chem. 27 (4), 589–603. 10.1016/j.bmc.2018.12.029 30600148

[B52] Liu Z. Yao Y. Kogiso M. Zheng B. Deng L. Qiu J. J. (2014). Inhibition of cancer-associated mutant isocitrate dehydrogenases: Synthesis, structure-activity relationship, and selective antitumor activity. J. Med. Chem. 57 (20), 8307–8318. 10.1021/jm500660f 25271760 PMC4207540

[B53] Losman J. A. Kaelin W. G. Jr. (2013). What a difference a hydroxyl makes: mutant IDH, (R)-2-hydroxyglutarate, and cancer. Genes Dev. 27 (8), 836–852. 10.1101/gad.217406.113 23630074 PMC3650222

[B54] Lu R. Wang J. Ren Z. Yin J. Wang Y. Cai L. (2019). A model system for studying the DNMT3A hotspot mutation (DNMT3A(R882)) demonstrates a causal relationship between its dominant-negative effect and leukemogenesis. Cancer Res. 79 (14), 3583–3594. 10.1158/0008-5472.Can-18-3275 31164355 PMC6897384

[B55] Ma D. L. Chan D. S. Leung C. H. (2013). Drug repositioning by structure-based virtual screening. Chem. Soc. Rev. 42 (5), 2130–2141. 10.1039/c2cs35357a 23288298

[B56] Ma R. Yun C. H. (2018). Crystal structures of pan-IDH inhibitor AG-881 in complex with mutant human IDH1 and IDH2. Biochem. Biophys. Res. Commun. 503 (4), 2912–2917. 10.1016/j.bbrc.2018.08.068 30131249

[B57] Ma T. Zou F. Pusch S. Yang L. Zhu Q. Xu Y. (2017). Design, synthesis and biological activity of 3-pyrazine-2-yl-oxazolidin-2-ones as novel, potent and selective inhibitors of mutant isocitrate dehydrogenase 1. Bioorg. Med. Chem. 25 (24), 6379–6387. 10.1016/j.bmc.2017.10.009 29089260

[B58] Machida Y. Nakagawa M. Matsunaga H. Yamaguchi M. Ogawara Y. Shima Y. (2020). A potent blood-brain barrier-permeable mutant IDH1 inhibitor suppresses the growth of glioblastoma with IDH1 mutation in a patient-derived orthotopic xenograft model. Mol. Cancer Ther. 19 (2), 375–383. 10.1158/1535-7163.Mct-18-1349 31727689

[B59] Madala H. R. Punganuru S. R. Arutla V. Misra S. Thomas T. J. Srivenugopal K. S. (2018). Beyond brooding on oncometabolic havoc in IDH-mutant gliomas and AML: Current and future therapeutic strategies. Cancers (Basel) 10 (2), E49. 10.3390/cancers10020049 29439493 PMC5836081

[B60] McMurry H. Fletcher L. Traer E. (2021). IDH inhibitors in AML-promise and pitfalls. Curr. Hematol. Malig. Rep. 16 (2), 207–217. 10.1007/s11899-021-00619-3 33939107

[B61] Megías-Vericat J. E. Ballesta-López O. Barragán E. Montesinos P. (2019). IDH1-mutated relapsed or refractory AML: Current challenges and future prospects. Blood Lymphat. Cancer 9, 19–32. 10.2147/blctt.S177913 31413655 PMC6663038

[B62] Mellinghoff I. K. Penas-Prado M. Peters K. B. Burris H. A. 3rd Maher E. A. Janku F. (2021). Vorasidenib, a dual inhibitor of mutant IDH1/2, in recurrent or progressive glioma; results of a first-in-human phase I trial. Clin. Cancer Res. 27 (16), 4491–4499. 10.1158/1078-0432.Ccr-21-0611 34078652 PMC8364866

[B63] Merk A. Bartesaghi A. Banerjee S. Falconieri V. Rao P. Davis M. I. (2016). Breaking cryo-EM resolution barriers to facilitate drug discovery. Cell 165 (7), 1698–1707. 10.1016/j.cell.2016.05.040 27238019 PMC4931924

[B64] Mohammad N. Wong D. Lum A. Lin J. Ho J. Lee C. H. (2020). Characterisation of isocitrate dehydrogenase 1/isocitrate dehydrogenase 2 gene mutation and the d-2-hydroxyglutarate oncometabolite level in dedifferentiated chondrosarcoma. Histopathology 76 (5), 722–730. 10.1111/his.14018 31609487

[B65] Nakagawa M. Nakatani F. Matsunaga H. Seki T. Endo M. Ogawara Y. (2019). Selective inhibition of mutant IDH1 by DS-1001b ameliorates aberrant histone modifications and impairs tumor activity in chondrosarcoma. Oncogene 38 (42), 6835–6849. 10.1038/s41388-019-0929-9 31406254

[B66] Norsworthy K. J. Luo L. Hsu V. Gudi R. Dorff S. E. Przepiorka D. (2019). FDA approval summary: Ivosidenib for relapsed or refractory acute myeloid leukemia with an isocitrate dehydrogenase-1 mutation. Clin. Cancer Res. 25 (11), 3205–3209. 10.1158/1078-0432.Ccr-18-3749 30692099

[B67] Okoye-Okafor U. C. Bartholdy B. Cartier J. Gao E. N. Pietrak B. Rendina A. R. (2015). New IDH1 mutant inhibitors for treatment of acute myeloid leukemia. Nat. Chem. Biol. 11 (11), 878–886. 10.1038/nchembio.1930 26436839 PMC5155016

[B68] Parsons D. W. Jones S. Zhang X. Lin J. C. Leary R. J. Angenendt P. (2008). An integrated genomic analysis of human glioblastoma multiforme. Science 321 (5897), 1807–1812. 10.1126/science.1164382 18772396 PMC2820389

[B69] Pellegatta S. Valletta L. Corbetta C. Patanè M. Zucca I. Riccardi Sirtori F. (2015). Effective immuno-targeting of the IDH1 mutation R132H in a murine model of intracranial glioma. Acta Neuropathol. Commun. 3, 4. 10.1186/s40478-014-0180-0 25849072 PMC4359524

[B70] Pirozzi C. J. Reitman Z. J. Yan H. (2013). Releasing the block: Setting differentiation free with mutant IDH inhibitors. Cancer Cell 23 (5), 570–572. 10.1016/j.ccr.2013.04.024 23680144 PMC4465106

[B71] Popovici-Muller J. Lemieux R. M. Artin E. Saunders J. O. Salituro F. G. Travins J. (2018). Discovery of AG-120 (ivosidenib): A first-in-class mutant IDH1 inhibitor for the treatment of IDH1 mutant cancers. ACS Med. Chem. Lett. 9 (4), 300–305. 10.1021/acsmedchemlett.7b00421 29670690 PMC5900343

[B72] Popovici-Muller J. Saunders J. O. Salituro F. G. Travins J. M. Yan S. Zhao F. (2012). Discovery of the first potent inhibitors of mutant IDH1 that lower tumor 2-HG *in vivo* . ACS Med. Chem. Lett. 3 (10), 850–855. 10.1021/ml300225h 24900389 PMC4025665

[B73] Radoul M. Hong D. Gillespie A. M. Najac C. Viswanath P. Pieper R. O. (2021). Early noninvasive metabolic biomarkers of mutant IDH inhibition in glioma. Metabolites 11 (2), 109. 10.3390/metabo11020109 33668509 PMC7917625

[B74] Reitman Z. J. Choi B. D. Spasojevic I. Bigner D. D. Sampson J. H. Yan H. (2012). Enzyme redesign guided by cancer-derived IDH1 mutations. Nat. Chem. Biol. 8 (11), 887–889. 10.1038/nchembio.1065 23001033 PMC3487689

[B75] Rohde J. M. Karavadhi S. Pragani R. Liu L. Fang Y. Zhang W. (2021). Discovery and optimization of 2H-1λ(2)-Pyridin-2-one inhibitors of mutant isocitrate dehydrogenase 1 for the treatment of cancer. J. Med. Chem. 64 (8), 4913–4946. 10.1021/acs.jmedchem.1c00019 33822623 PMC8968748

[B76] Rohle D. Popovici-Muller J. Palaskas N. Turcan S. Grommes C. Campos C. (2013). An inhibitor of mutant IDH1 delays growth and promotes differentiation of glioma cells. Science 340 (6132), 626–630. 10.1126/science.1236062 23558169 PMC3985613

[B77] Scagliola A. Mainini F. Cardaci S. (2020). The tricarboxylic acid cycle at the crossroad between cancer and immunity. Antioxid. Redox Signal. 32 (12), 834–852. 10.1089/ars.2019.7974 31847530

[B78] Schumacher T. Bunse L. Pusch S. Sahm F. Wiestler B. Quandt J. (2014). A vaccine targeting mutant IDH1 induces antitumour immunity. Nature 512 (7514), 324–327. 10.1038/nature13387 25043048

[B79] Sjöblom T. Jones S. Wood L. D. Parsons D. W. Lin J. Barber T. D. (2006). The consensus coding sequences of human breast and colorectal cancers. Science 314 (5797), 268–274. 10.1126/science.1133427 16959974

[B80] Stein E. M. DiNardo C. D. Fathi A. T. Mims A. S. Pratz K. W. Savona M. R. (2021). Ivosidenib or enasidenib combined with intensive chemotherapy in patients with newly diagnosed AML: a phase 1 study. Blood 137 (13), 1792–1803. 10.1182/blood.2020007233 33024987 PMC8020270

[B81] Su Y. T. Phan F. P. Wu J. (2018). Perspectives on IDH mutation in diffuse gliomas. Trends Cancer 4 (9), 605–607. 10.1016/j.trecan.2018.06.006 30149878 PMC10335607

[B82] Tabata M. M. Chase M. Kwong B. Y. Novoa R. A. Fernandez-Pol S. (2020). Differentiation syndrome during ivosidenib treatment with immunohistochemistry showing isocitrate dehydrogenase R132H mutation. J. Cutan. Pathol. 47 (11), 1042–1045. 10.1111/cup.13780 32588467

[B83] Tobinick E. L. (2009). The value of drug repositioning in the current pharmaceutical market. Drug News Perspect. 22 (2), 119–125. 10.1358/dnp.2009.22.2.1303818 19330170

[B84] Tong J. B. Bian S. Zhang X. Luo D. (2021). QSAR analysis of 3-pyrimidin-4-yl-oxazolidin-2-one derivatives isocitrate dehydrogenase inhibitors using Topomer CoMFA and HQSAR methods. Mol. Divers. 26, 1017–1037. 10.1007/s11030-021-10222-6 33974175

[B85] Wang P. Wu J. Ma S. Zhang L. Yao J. Hoadley K. A. (2015). Oncometabolite D-2-hydroxyglutarate inhibits ALKBH DNA repair enzymes and sensitizes IDH mutant cells to alkylating agents. Cell Rep. 13 (11), 2353–2361. 10.1016/j.celrep.2015.11.029 26686626 PMC4694633

[B86] Wang Q. Shao X. Leung E. L. H. Chen Y. Yao X. (2021). Selectively targeting individual bromodomain: Drug discovery and molecular mechanisms. Pharmacol. Res. 172, 105804. 10.1016/j.phrs.2021.105804 34450309

[B87] Wang Y. Tang S. Lai H. Jin R. Long X. Li N. (2020). Discovery of novel IDH1 inhibitor through comparative structure-based virtual screening. Front. Pharmacol. 11, 579768. 10.3389/fphar.2020.579768 33262701 PMC7686577

[B88] Weller M. Wick W. Aldape K. Brada M. Berger M. Pfister S. M. (2015). Glioma. Nat. Rev. Dis. Prim. 1, 15017. 10.1038/nrdp.2015.17 27188790

[B89] Wenger K. J. Richter C. Burger M. C. Urban H. Kaulfuss S. Harter P. N. (2020). Non-invasive measurement of drug and 2-HG signals using (19)F and (1)H MR spectroscopy in brain tumors treated with the mutant IDH1 inhibitor BAY1436032. Cancers (Basel) 12 (11), E3175. 10.3390/cancers12113175 33138036 PMC7692790

[B90] Wick A. Bähr O. Schuler M. Rohrberg K. Chawla S. P. Janku F. (2021). Phase I assessment of safety and therapeutic activity of BAY1436032 in patients with IDH1-mutant solid tumors. Clin. Cancer Res. 27 (10), 2723–2733. 10.1158/1078-0432.Ccr-20-4256 33622704

[B91] Wu F. Cheng G. Yao Y. Kogiso M. Jiang H. Li X. N. (2018). Inhibition of mutated isocitrate dehydrogenase 1 in cancer. Med. Chem. 14 (7), 715–724. 10.2174/1573406414666180524093659 29792149 PMC6205205

[B92] Wu F. Jiang H. Zheng B. Kogiso M. Yao Y. Zhou C. (2015). Inhibition of cancer-associated mutant isocitrate dehydrogenases by 2-thiohydantoin compounds. J. Med. Chem. 58 (17), 6899–6908. 10.1021/acs.jmedchem.5b00684 26280302 PMC4567406

[B93] Xu S. Tang L. Li X. Fan F. Liu Z. (2020). Immunotherapy for glioma: Current management and future application. Cancer Lett. 476, 1–12. 10.1016/j.canlet.2020.02.002 32044356

[B94] Yang B. Zhong C. Peng Y. Lai Z. Ding J. (2010). Molecular mechanisms of "off-on switch" of activities of human IDH1 by tumor-associated mutation R132H. Cell Res. 20 (11), 1188–1200. 10.1038/cr.2010.145 20975740

[B95] Yang Z. Jiang B. Wang Y. Ni H. Zhang J. Xia J. (2017). 2-HG inhibits necroptosis by stimulating DNMT1-dependent hypermethylation of the RIP3 promoter. Cell Rep. 19 (9), 1846–1857. 10.1016/j.celrep.2017.05.012 28564603

[B96] Yasutake Y. Watanabe S. Yao M. Takada Y. Fukunaga N. Tanaka I. (2002). Structure of the monomeric isocitrate dehydrogenase: evidence of a protein monomerization by a domain duplication. Structure 10 (12), 1637–1648. 10.1016/s0969-2126(02)00904-8 12467571

[B97] Ye D. Guan K. L. Xiong Y. (2018). Metabolism, activity, and targeting of D- and L-2-hydroxyglutarates. Trends Cancer 4 (2), 151–165. 10.1016/j.trecan.2017.12.005 29458964 PMC5884165

[B98] Zhang N. Zheng B. Yao X. Huang X. Du J. Shen Y. (2021). Identification and characterization of a novel mutant isocitrate dehydrogenase 1 inhibitor for glioma treatment. Biochem. Biophys. Res. Commun. 551, 38–45. 10.1016/j.bbrc.2021.02.112 33714758

[B99] Zhao Q. Manning J. R. Sutton J. Costales A. Sendzik M. Shafer C. M. (2018). Optimization of 3-Pyrimidin-4-yl-oxazolidin-2-ones as orally bioavailable and brain penetrant mutant IDH1 inhibitors. ACS Med. Chem. Lett. 9 (7), 746–751. 10.1021/acsmedchemlett.8b00182 30034612 PMC6047033

[B100] Zheng B. Yao Y. Liu Z. Deng L. Anglin J. L. Jiang H. (2013). Crystallographic investigation and selective inhibition of mutant isocitrate dehydrogenase. ACS Med. Chem. Lett. 4 (6), 542–546. 10.1021/ml400036z 23795241 PMC3686309

[B101] Zheng Q. Tang S. Fu X. Chen Z. Ye Y. Lan X. (2017). Discovery and structure-activity-relationship study of novel conformationally restricted indane analogues for mutant isocitric dehydrogenase 1 (IDH1) inhibitors. Bioorg. Med. Chem. Lett. 27 (23), 5262–5266. 10.1016/j.bmcl.2017.10.029 29079473

[B102] Zheng, M M. Tang R. Deng Y. Yang K. Chen L. Li H. (2018). Steroids from Ganoderma sinense as new natural inhibitors of cancer-associated mutant IDH1. Bioorg. Chem. 79, 89–97. 10.1016/j.bioorg.2018.04.016 29738972

[B103] Zheng, Q Q. Chen Z. Wan H. Tang S. Ye Y. Xu Y. (2018). Discovery and structure-activity-relationship study of novel imidazole cyclopropyl amine analogues for mutant isocitric dehydrogenase 1 (IDH1) inhibitors. Bioorg. Med. Chem. Lett. 28 (23-24), 3808–3812. 10.1016/j.bmcl.2018.07.002 30413349

[B104] Zhu A. X. Macarulla T. Javle M. M. Kelley R. K. Lubner S. J. Adeva J. (2021). Final overall survival efficacy results of ivosidenib for patients with advanced cholangiocarcinoma with IDH1 mutation: The phase 3 randomized clinical ClarIDHy trial. JAMA Oncol. 7, 1669–1677. 10.1001/jamaoncol.2021.3836 34554208 PMC8461552

[B105] Zhu C. Ma Y. Xu X. P. (2014). Research progress on genes associated with transformation of myelodysplastic syndromes to acute myeloid leukemia. Zhongguo Shi Yan Xue Ye Xue Za Zhi 22 (3), 873–878. 10.7534/j.issn.1009-2137.2014.03.057 24989313

[B106] Zou F. Pusch S. Hua J. Ma T. Yang L. Zhu Q. (2018). Identification of novel allosteric inhibitors of mutant isocitrate dehydrogenase 1 by cross docking-based virtual screening. Bioorg. Med. Chem. Lett. 28 (3), 388–393. 10.1016/j.bmcl.2017.12.030 29290542

